# Necroptosis of macrophage is a key pathological feature in biliary atresia via GDCA/S1PR2/ZBP1/p-MLKL axis

**DOI:** 10.1038/s41419-023-05615-4

**Published:** 2023-03-01

**Authors:** Shen Yang, Na Chang, Weiyang Li, Ting Yang, Renmin Xue, Jing Liu, Li Zhang, Xingfeng Yao, Yajun Chen, Huanmin Wang, Lin Yang, Jinshi Huang, Liying Li

**Affiliations:** 1 Department of Cell Biology, Municipal Laboratory for Liver Protection and Regulation of Regeneration, Capital Medical University, Beijing, 100069 China; 2 Department of Neonatal Surgery, Beijing Children’s Hospital, Capital Medical University, National Center for Children’s Health, Beijing, 100045 China; 3 Institute of Precision Medicine, the Ninth People’s Hospital, Shanghai Jiao Tong University School of Medicine, Shanghai, 200125 China; 4 Department of Pathology, Beijing Children’s Hospital, Capital Medical University, National Center for Children’s Health, Beijing, 100045 China; 5 Department of General Surgery, Beijing Children’s Hospital, Capital Medical University, National Center for Children’s Health, Beijing, 100045 China; 6 Department of Surgical Oncology, Beijing Children’s Hospital, Capital Medical University, National Center for Children’s Health, Beijing, 100045 China

**Keywords:** Chronic inflammation, Liver fibrosis

## Abstract

Biliary atresia (BA) is a severe inflammatory and fibrosing neonatal cholangiopathy disease characterized by progressive obstruction of extrahepatic bile ducts, resulting in cholestasis and progressive hepatic failure. Cholestasis may play an important role in the inflammatory and fibrotic pathological processes, but its specific mechanism is still unclear. Necroptosis mediated by Z-DNA-binding protein 1 (ZBP1)/phosphorylated-mixed lineage kinase domain-like pseudokinase (p-MLKL) is a prominent pathogenic factor in inflammatory and fibrotic diseases, but its function in BA remains unclear. Here, we aim to determine the effect of macrophage necroptosis in the BA pathology, and to explore the specific molecular mechanism. We found that necroptosis existed in BA livers, which was occurred in liver macrophages. Furthermore, this process was mediated by ZBP1/p-MLKL, and the upregulated expression of ZBP1 in BA livers was correlated with liver fibrosis and prognosis. Similarly, in the bile duct ligation (BDL) induced mouse cholestatic liver injury model, macrophage necroptosis mediated by ZBP1/p-MLKL was also observed. In vitro, conjugated bile acid-glycodeoxycholate (GDCA) upregulated ZBP1 expression in mouse bone marrow-derived monocyte/macrophages (BMDMs) through sphingosine 1-phosphate receptor 2 (S1PR2), and the induction of ZBP1 was a prerequisite for the enhanced necroptosis. Finally, after selectively knocking down of macrophage *S1pr2* in vivo, ZBP1/p-MLKL-mediated necroptosis was decreased, and further collagen deposition was markedly attenuated in BDL mice. Furthermore, macrophage *Zbp1* or *Mlkl* specific knockdown also alleviated BDL-induced liver injury/fibrosis. In conclusion, GDCA/S1PR2/ZBP1/p-MLKL mediated macrophage necroptosis plays vital role in the pathogenesis of BA liver fibrosis, and targeting this process may represent a potential therapeutic strategy for BA.

## Introduction

Biliary atresia (BA) is a severe neonatal cholangiopathy characterized by a progressive fibroinflammatory obstruction of extrahepatic bile ducts resulting in cholestasis and progressive hepatic failure [1]. Although hepatic portoenterostomy (Kasai procedure) can re-establish extrahepatic bile drainage, about 70% of patients with progressive fibrosis and cirrhosis will eventually require liver transplantation for long-term survival [2]. However, there is no effective way to prevent the progressive liver inflammation and fibrosis that occur even after successful Kasai surgery. Cholestasis is a typical pathological feature in BA, and it is also an important feature of various liver diseases, such as primary biliary cholangitis (PBC) and primary sclerosing cholangitis (PSC) [3]. In the pathological processes of BA, cholestasis may play an important role, especially the accumulation of conjugated bile acids may lead to the activation of inflammatory pathways, liver injury and fibrosis [4]. Although previous studies have suggested that cholestasis is a cause of BA fibrosis, the specific molecular mechanism is still unclear.

Necroptosis is a programmed form of necrotic cell death, causes cell membrane rupture and triggers inflammation through the release of intracellular contents, which acts as damage-associated molecular patterns (DAMPs) [5–7]. As a kind of programmed cell death, necroptosis is regulated by a series of related molecules, such as phosphorylated-mixed lineage kinase domain-like pseudokinase (p-MLKL). Current studies suggest that p-MLKL-mediated necroptosis plays a role in a variety of diseases, such as ischemia-reperfusion injury, age-related macular degeneration, atherosclerosis, inflammatory bowel disease, and allograft rejection [8, 9]. As far as in liver diseases, necroptosis, which triggers hepatic regeneration, inflammation, and fibrogenesis [10], has been proved as a player of chronic liver diseases, including drug-induced liver injury [11], autoimmune hepatitis [12], nonalcoholic steatohepatitis (NASH) [13, 14], and non-alcoholic fatty liver disease [15]. In recent years, the role of macrophage necroptosis in liver inflammation and fibrosis has been gradually paid attention, but current studies are mainly limited to NASH [14] and bacterial infection [16]. However, it is still unclear whether necroptosis occurs in BA. On the other hand, the mechanism underlying necroptosis has been studied in previous studies. Z-DNA-binding protein 1 (ZBP1) has been proved as one of the most important drivers of necroptosis [17]. Since it is also unclear whether ZBP1 is involved in BA, the following questions need to be further studied and answered: does necroptosis occur in liver cells (especially macrophages) during BA process? If the answer is yes, is the necroptosis in BA mediated by ZBP1?

Hepatic macrophages, which are composed by resident Kupffer cells (KCs) and recruited bone marrow-derived monocyte/macrophages (BMDMs), are important players of BA. In BA patient livers or mouse model, macrophages are recruited to the injured liver by cholangiocyte-secreted chemokine (C-C motif) ligand 2 and aggravate bile duct injury [18]. In previous studies, macrophages are reported to mediate liver inflammation mainly by activating into pro-inflammatory subtype and secreting inflammatory factors. For example, macrophages aggravate cholestatic liver inflammation and fibrosis by secreting lncRNA-H19 [19] and inducing neutrophil chemotaxis by secreting macrophage inflammatory protein 2 [20]. Our previous studies have also shown that in bile duct ligation (BDL)-induced cholestatic liver injury, a large number of BMDMs recruit to the site of injured liver and differentiate to pro-inflammatory macrophages, which promote the occurrence and development of liver inflammation, fibrosis and cirrhosis [21–23]. Besides these, recent studies have also shown that necroptosis of macrophages significantly promote the progression of liver inflammation and fibrosis [14]. However, the role of macrophage necroptosis in cholestatic liver diseases (especially in BA) remains largely unknown.

In this study, we aim to explore the pathological features and regulatory mechanisms of BA cholestatic liver fibrosis, and further to find potential therapeutic strategies. We found that ZBP1/p-MLKL-mediated necroptosis existed in BA livers, especially in macrophages. ZBP1 expression was upregulated in BA livers and the abnormal increased expression of ZBP1 in BA livers was correlated with liver fibrosis and prognosis. Furthermore, the similar pathological features were observed in the livers of BDL-induced mouse cholestatic liver injury. In vitro, conjugated bile acid-glycodeoxycholate (GDCA) upregulated ZBP1 expression in mouse BMDMs through sphingosine 1-phosphate receptor 2 (S1PR2), and the GDCA-induced upregulation of ZBP1 was a basis for the enhanced necroptosis. In BDL-induced liver fibrosis, selectively knocking down of macrophage *S1pr2*, *Zbp1* or *Mlkl* blocked S1PR2/ZBP1/p-MLKL axis. Blockage of S1PR2/ZBP1/p-MLKL axis caused the decrease of necroptosis, and importantly, the attenuation of liver fibrosis. These data suggest that macrophage necroptosis mediated by GDCA/S1PR2/ZBP1/p-MLKL plays a significant role in the pathogenesis of cholestasis-induced liver fibrosis, and may represent a potential therapeutic strategy for BA.

## Results

### Necroptosis mediated by ZBP1/p-MLKL occurred in BA livers

**Fig. 1:**
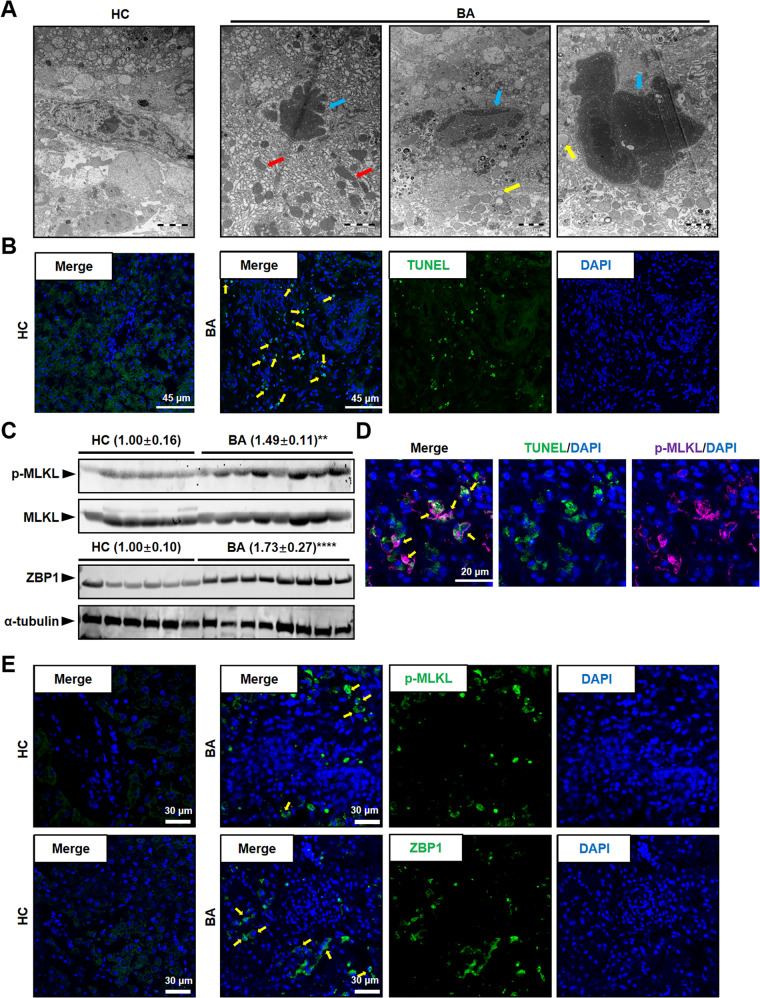
Necroptosis existed in BA livers. **A** Representative TEM images in normal adjacent non-tumor livers (HC) and biliary atresia (BA) livers. Red arrows indicate enlarged mitochondria, blue arrows indicate swollen nucleus, and yellow arrows indicate enlarged vacuoles. Scale bar: 2 μm. **B** Representative TUNEL staining images in HC and BA livers. Yellow arrows: TUNEL^+^ cells. Scale bar: 45 μm. **C** Western blot analysis for p-MLKL and ZBP1 in the liver tissues from BA (*n* = 8) and HC (*n* = 6). Blots of p-MLKL were normalized to MLKL, and blots of ZBP1 were normalized to α-tubulin. **D** Representative image of TUNEL (green) and p-MLKL (pink) staining in BA livers. Yellow arrows: TUNEL^+^p-MLKL^+^ cells. Scale bar: 20 μm. **E** Representative images of p-MLKL (green) or ZBP1 (green) staining. The nuclei were stained with DAPI (blue). Yellow arrows: p-MLKL^+^ or ZBP1^+^ cells. Scale bar: 30 μm. Data are presented as the mean ± SEM. **p < 0.01, ****p < 0.0001 (versus control).

**Fig. 2:**
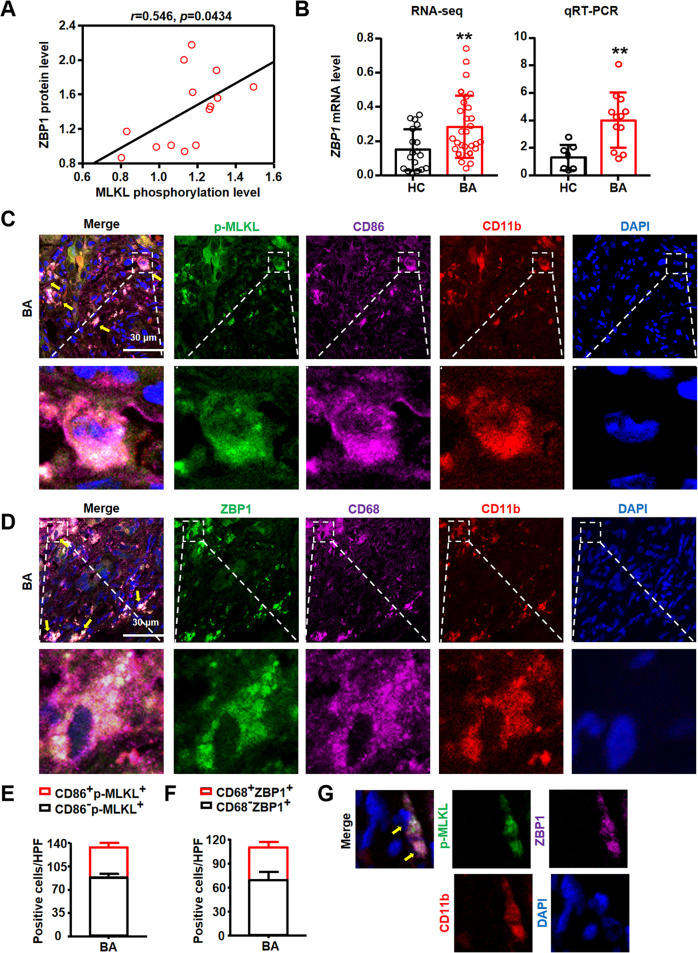
ZBP1/p-MLKL mediated necroptosis existed in macrophages in BA livers. **A** The correlation between ZBP1 and p-MLKL in human liver tissues. **B** Hepatic *ZBP1* mRNA level detected by RNA sequencing and qRT-PCR were compared between biliary atresia (BA, RNA-seq: *n* = 31, qRT-PCR: *n* = 12) and normal adjacent non-tumor livers (HC, RNA-seq: *n* = 20, qRT-PCR: *n* = 7). **C** Representative images of p-MLKL (green), CD86 (pink) and CD11b (red) staining. The nuclei were stained with DAPI (blue). Yellow arrows: p-MLKL^+^CD86^+^CD11b^+^ cells. Scale bar: 30 μm. **D** Representative images of ZBP1 (green), CD68 (pink) and CD11b (red). The nuclei were stained with DAPI (blue). Yellow arrows: ZBP1^+^CD68^+^CD11b^+^ cells. Scale bar: 30 μm. **E**, **F** Quantitative results of p-MLKL^+^(**E**) and ZBP1^+^ (**F**) macrophage number in BA livers. **G** Representative images of p-MLKL (green), ZBP1 (pink) and CD11b (red) staining in BA livers. The nuclei were stained with DAPI (blue). Yellow arrows: p-MLKL^+^ZBP1^+^CD11b^+^ cells. Data are presented as the mean ± SEM. **p < 0.01 (versus control).

To study the pathological characters of BA liver, BA patient liver sections were collected and performed H&E staining. The results showed markedly immune cell infiltration in BA livers (Supplementary Fig. 1A). In the fibrotic niche of BA livers, a typical visual pattern of necrotic cell death was clearly identified through the transmission electron microscope (TEM) images, including swollen nucleus and cells, disintegrated cell membrane and nuclear membrane, enlarged mitochondria, and appearance of globular vacuoles (Fig. 1A). Furthermore, we observed a large number of TUNEL-positive necrotic or apoptotic non-parenchymal cells (NPCs) in the fibrotic niche of BA livers, while TUNEL-positive cells were almost undetectable in control subjects (Fig. 1B). To further confirm whether the cell death occurred in BA liver is necroptosis, we examined the level of phosphorylated MLKL (S358), the marker of necroptosis, using western blot and immunofluorescence (IF) studies. The results showed that the level of MLKL phosphorylation was obviously higher in BA livers than control subjects (Fig. 1C, E). At the same time, TUNEL positive cells were also positive for phosphorylated MLKL (p-MLKL), indicating liver cells occurred p-MLKL-mediated necroptosis in BA (Fig. 1D). As far as necroptosis mediated by ZBP1/p-MLKL was reported in former studies [17], we detected the expression of ZBP1 in BA livers. The western blot and IF results showed that ZBP1 level was higher in BA livers than control subjects (Fig. 1C, E), and the protein levels of ZBP1 were positively correlated with p-MLKL (Fig. 2A). Otherwise, RNA sequencing and qRT-PCR results showed that *ZBP1* mRNA level was much higher in BA patients than in control subjects (Fig. 2B). Taken together, these results suggested that necroptosis mediated by ZBP1/p-MLKL occurred in BA livers, especially in NPCs.

### Necroptosis mediated by ZBP1/p-MLKL occurred in BA liver macrophages

The above results showed that NPCs in the fibrotic niche in BA livers occurred necroptosis. As one of the main components of liver NPCs, macrophages localized in the fibrotic niche play an important role in BA via promoting liver inflammation and fibrosis [19]. We next examined whether necroptosis occurred in macrophages in BA livers. IF staining was performed using p-MLKL (S358) (the marker of necroptosis), CD86 and CD11b. The result showed that cells with positive p-MLKL staining were also positive for CD86 and CD11b staining (Fig. 2C). Furthermore, we detected the localization of ZBP1 through IF staining, and the results showed that significant numbers of ZBP1^+^ cells were also positive for CD68 and CD11b in BA livers (Fig. 2D), which was consistent with the results of p-MLKL. Furthermore, quantitative results showed that 44% ± 1% of p-MLKL^+^ cells (necroptosis) were macrophages (CD86^+^p-MLKL^+^, Fig. 2E), and similarly, 42% ± 4% ZBP1^+^cells were also macrophages (CD68^+^ZBP1^+^, Fig. 2F). We also studied whether p-MLKL was co-localized with ZBP1 in BA livers. The results of IF showed the co-localization of p-MLKL and ZBP1 in CD11b^+^ cells (Fig. 2G). In conclusion, these results confirmed that ZBP1/p-MLKL-dependent necroptosis occurred in macrophages in BA livers.

### Necroptosis mediated by ZBP1/p-MLKL occurred in BMDMs in BDL livers

**Fig. 3:**
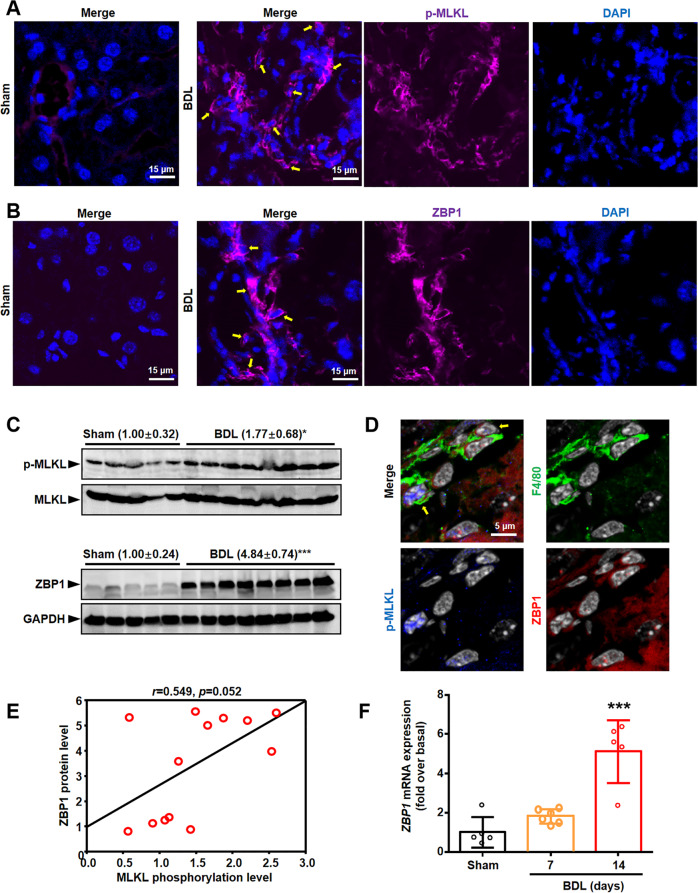
Necroptosis existed in BDL livers. **A** Representative images of p-MLKL (pink) staining. The nuclei were stained with DAPI (blue). Yellow arrows: p-MLKL^+^ cells. Scale bar: 15 μm. **B** Representative images of ZBP1 (pink) staining. The nuclei were stained with DAPI (blue). Yellow arrows: ZBP1^+^ cells. Scale bar: 15 μm. **C** Western blot analysis for p-MLKL and ZBP1 in the liver tissues from BDL (*n* = 8) and Sham mice (*n* = 5). Bolts of p-MLKL were normalized to MLKL. Blots of ZBP1 were normalized to GAPDH. **D** Representative images of F4/80 (green), ZBP1 (red) and p-MLKL (blue) staining in BDL livers. The nuclei were stained with DAPI (white). Yellow arrows: p-MLKL^+^ZBP1^+^F4/80^+^ cells. **E** The correlation between ZBP1 and p-MLKL levels in mice liver tissues. **F** mRNA level of *Zbp1* in mice livers was measured by qRT-PCR. Data are presented as the mean ± SEM. *p < 0.05, ***p < 0.001 (versus control).

To further examine the role of necroptosis in BA, especially in cholestasis-induced liver fibrosis, BDL mouse model, a classic experimental model of cholestasis and secondary biliary fibrosis, was used. We found that p-MLKL and ZBP1 protein levels increased in BDL mice livers through western blot and IF analysis (Fig. 3A–C). Furthermore, the protein level of ZBP1 was positively correlated with p-MLKL level (Fig. 3E). Previous studies have reported that receptor-interacting serine/threonine-protein kinase 1 (RIPK1) and RIPK3, which are the inducer of MLKL phosphorylation, interact with ZBP1 to induce necroptosis [17, 24]. We also studied the activation of RIPK1 and RIPK3 in BDL mouse model. The results of western blot showed that p-RIPK1 and p-RIPK3 levels were also increased in BDL livers (Supplementary Fig. 1B). qRT-PCR analysis also showed that mRNA level of *Zbp1* was increased in BDL mice than that in Sham mice (Fig. 3F). Moreover, in consistent with BA livers, we also detected p-MLKL^+^ZBP1^+^ macrophages (F4/80^+^ cells) in BDL-induced mouse liver fibrosis (Fig. 3D).

**Fig. 4:**
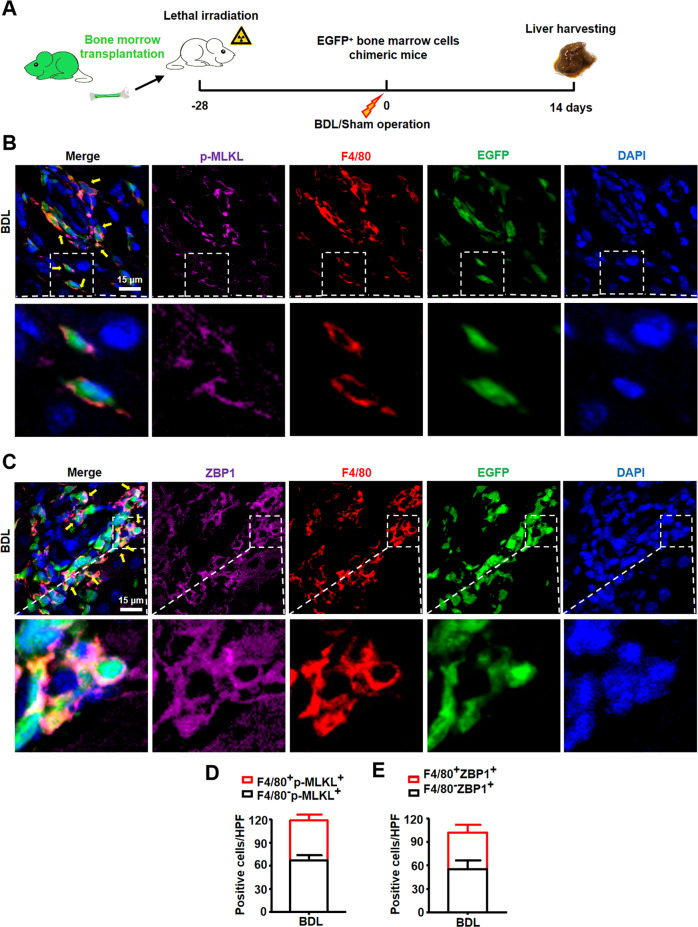
ZBP1/p-MLKL mediated necroptosis existed in BMDMs in BDL livers. **A** The schedule of mouse model. **B** Representative images of p-MLKL (pink) and F4/80 (red) staining in BDL livers. EGFP (green) indicated bone marrow-derived cells. The nuclei were stained with DAPI (blue). Yellow arrows: p-MLKL^+^F4/80^+^EGFP^+^ cells. Scale bar: 15 μm. **C** Representative images of ZBP1 (pink) and F4/80 (red) staining in BDL livers. EGFP (green) indicated bone marrow-derived cells. The nuclei were stained with DAPI (blue). Yellow arrows: ZBP1^+^F4/80^+^EGFP^+^ cells. Scale bar: 15 μm. **D**, **E** Quantitative results of p-MLKL^+^ (**D**) and ZBP1^+^ (**E**) macrophage number in BDL livers. Data are presented as the mean ± SEM.

It has been reported that there are two kinds of macrophages in damaged liver, resident KCs and recruited BMDMs. To further confirm which kind of macrophages occurred necroptosis after BDL injury, mice were lethally irradiated and received whole bone marrow cell transplants from enhanced green fluorescent protein (EGFP) transgenic mice, followed by BDL or sham operation. At two weeks after BDL or sham operation, livers were harvested (Fig. 4A). The results of IF staining showed that cells with p-MLKL positive staining were also positive for F4/80 (mouse macrophage marker) and EGFP (represented cells of bone marrow origin) (Fig. 4B). Similarly, significant numbers of ZBP1^+^ cells were also positive for F4/80 and EGFP in BDL livers (Fig. 4C). Quantitative results showed that 48% ± 3% of p-MLKL^+^ cells (necroptosis) were macrophages (F4/80^+^p-MLKL^+^, Fig. 4D), while 47% ± 2% of ZBP1^+^ cells were macrophages (F4/80^+^ZBP1^+^, Fig. 4E). Thus, these results suggested that ZBP1/p-MLKL-dependent necroptosis occurs in macrophages, especially in BMDMs in BDL livers.

### *Zbp1* was dominantly highly expressed in BMDMs after BDL injury through single-cell RNA sequencing (scRNA-seq) analysis

**Fig. 5:**
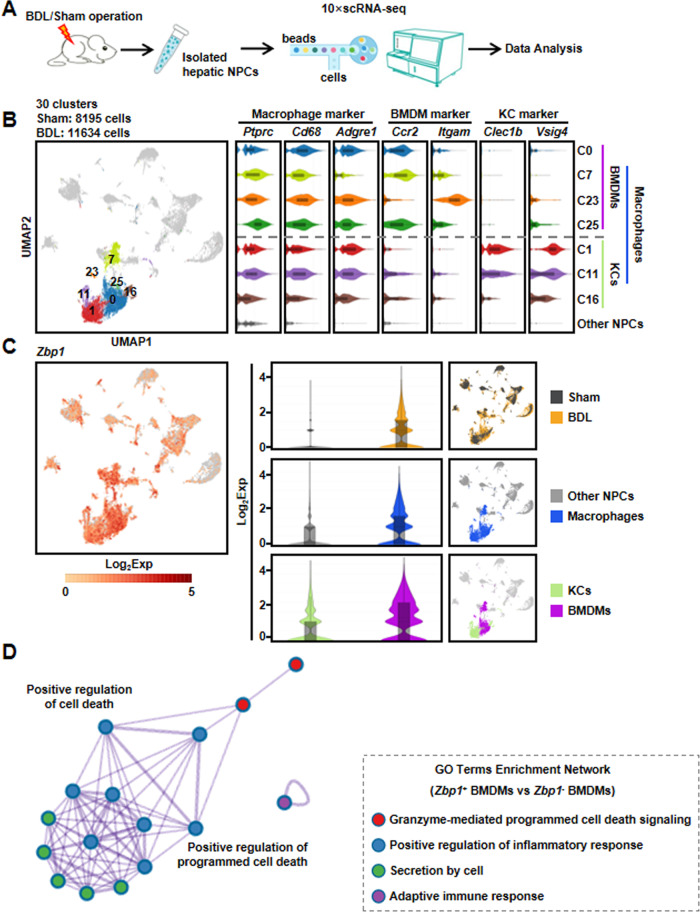
scRNA-seq analysis showed that *Zbp1* was dominantly and highly expressed in BMDMs after BDL injury. **A** Schematic diagram of the experimental design for scRNA-seq. **B** Uniform manifold approximation and projection (UMAP) plot depicted 19,829 cells representing NPC clusters (left panel). Violin plots showed marker gene expressions across macrophage, bone marrow derived macrophage (BMDM), and Kupffer cell (KC) clusters (right panel). **C** Violin plots showed the expression of *Zbp1* in each cell clusters. **D** Gene Ontology (GO) analysis was performed based on the differential expressed genes between ZBP1^+^ and ZBP1^−^ BMDMs in scRNA-seq.

To further identify the cell types with increased expression of *Zbp1* in BDL, we isolated 19,829 NPCs from one BDL (11,634 NPCs) and one Sham mouse (8195 NPCs) to perform scRNA-seq (Fig. 5A), respectively. After data processing, NPCs were partitioned into 30 major clusters based on the shared gene expression features (Fig. 5B). According to the expression of known macrophage markers (*Ptprc, Cd68*, and *Adgre1*), we identified seven clusters of macrophages (named as C0, C1, C7, C11, C16, C23, and C25). Furthermore, four clusters of BMDMs (gene markers of *Ccr2* and *Itgam*) and three clusters of KCs (gene markers of *Clec1b* and *Vsig4*) were obtained, respectively (Fig. 5B). Next, we focused on the expression pattern of *Zbp1* in different cell clusters, and the result showed that BDL mouse contained significantly increased proportions of *Zbp1*-expressing cells compared with those of Sham mouse. More importantly, *Zbp1* was dominantly highly expressed in macrophages, especially in BMDMs (Fig. 5C). To further explore the specific functions of ZBP1^+^ BMDMs after BDL injury, the differential expressed genes between ZBP1^+^ and ZBP1^−^ BMDMs of the scRNA-seq were used to perform Gene Ontology (GO) analysis. The GO terms included granzyme-mediated programmed cell death signaling, positive regulation of inflammatory response, secretion by cell, and adaptive immune response (Fig. 5D). These results further indicated that ZBP1^+^ BMDMs played vital roles in mediating cell death and inflammation in BDL-induced liver injury.

### GDCA upregulated ZBP1 expression in mouse BMDMs through S1PR2

**Fig. 6:**
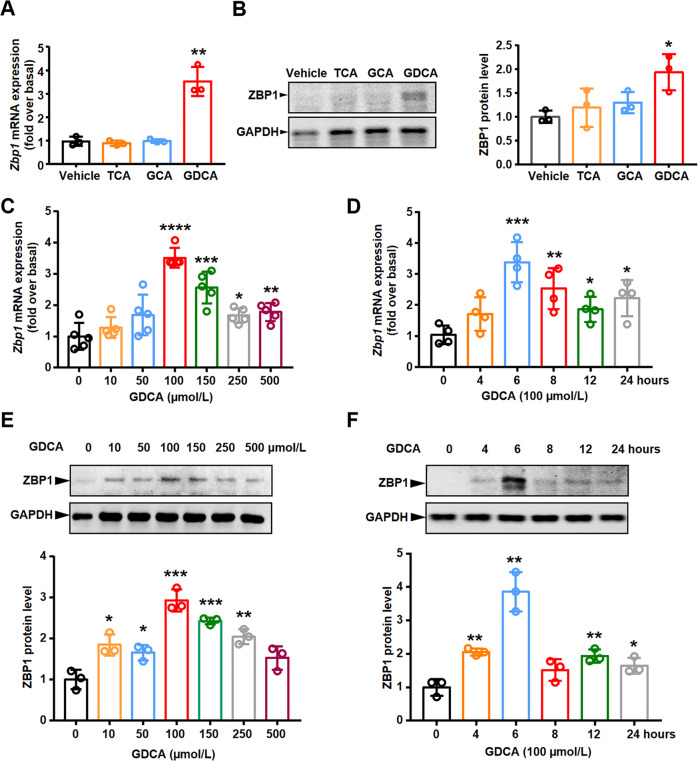
GDCA upregulated ZBP1 expression in mouse BMDMs in a time- and dose-dependent manner. **A**, **B** ZBP1 mRNA (**A**) and protein (**B**) levels were measured by qRT-PCR and western blot in BMDMs treated with 100 μmol/L sodium taurocholate hydrate (TCA), glycocholic acid hydrate (GCA), and sodium glycodeoxycholate (GDCA) for 6 h, respectively. **C** *Zbp1* mRNA level was measured by qRT-PCR in mouse BMDMs treated with the indicated concentrations of GDCA for 6 h. **D** *Zbp1* mRNA level was measured by qRT-PCR in mouse BMDMs treated with 100 μmol/L GDCA for different time points. **E**, **F** ZBP1 protein level was measured in mouse BMDMs treated with the indicated concentrations of GDCA (**E**) or different time points (**F**). All results were confirmed by at least three independent experiments. Data are presented as the mean ± SEM. *p < 0.05, **p < 0.01, ***p < 0.001, ****p < 0.0001 (versus control).

Former studies have shown that the conjugated bile acid levels are significantly elevated in liver and plasma of BA patients, causing by disrupted bile flow from the liver to the intestine [25–28]. Similarly, in the mouse BDL model, more than 99% of bile acids in liver and serum were conjugated bile acids, especially taurocholate hydrate (TCA), glycocholic acid hydrate (GCA), and GDCA [29, 30]. To further explore whether bile acids induced increased ZBP1 expression in BA and BDL livers, BMDMs were treated with different conjugated bile acids in vitro. Interestingly, we found that the mRNA and protein levels of ZBP1 were exclusively induced by GDCA (Fig. 6A, B). Furthermore, GDCA induced the expression of ZBP1 in a time- and dose-dependent manner (Fig. 6C–F).

**Fig. 7:**
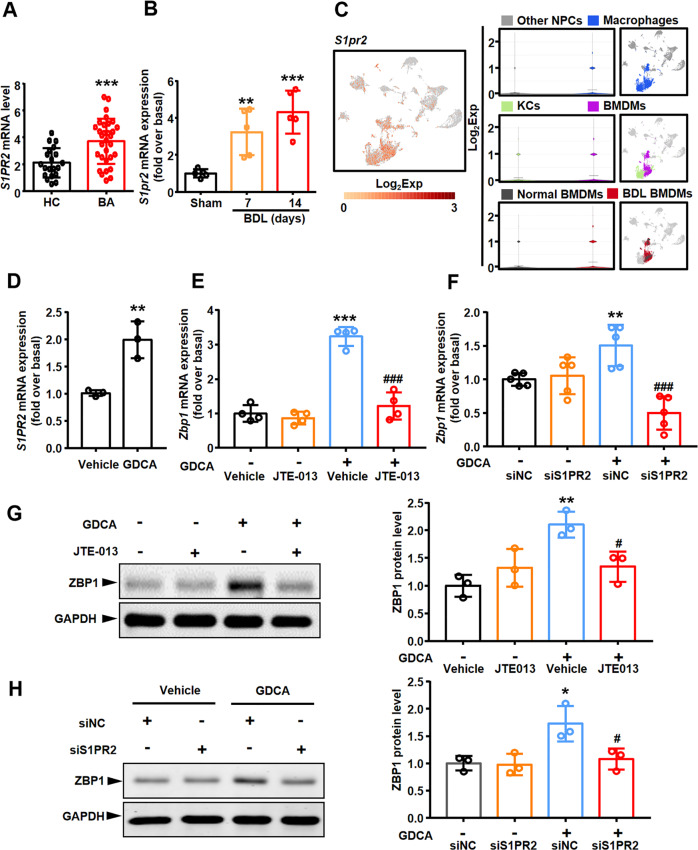
GDCA increased ZBP1 expression in mouse BMDMs through S1PR2. **A** Hepatic *S1PR2* mRNA level detected by RNA sequencing was compared between biliary atresia (BA, *n* = 31) and normal adjacent non-tumor livers (HC, *n* = 20). **B** mRNA level of *S1pr2* in mouse livers was measured by qRT-PCR. **C** Violin plots showed *S1pr2* expression of each cell clusters in scRNA-seq data. **D** *S1pr2* mRNA level was measured by qRT-PCR in mouse BMDMs treated with 100 μmol/L sodium glycodeoxycholate (GDCA) for 6 h. **E** Mouse BMDMs were pretreated with S1PR2 antagonist JTE-013 (10 μmol/L) for 1 h, followed by 100 μmol/L GDCA for 6 h, and *Zbp1* mRNA level was measured by qRT-PCR. **F** Effect of *S1pr2* siRNA on *Zbp1* mRNA expression in response to 100 μmol/L GDCA for 6 h. **G** ZBP1 protein level was measured in mouse BMDMs pretreated with JTE-013, followed by GDCA. **H** Effect of *S1pr2* siRNA on ZBP1 protein expression in response to 100 μmol/L GDCA for 6 h. All results were confirmed by at least three independent experiments. Data are presented as the mean ± SEM. *p < 0.05, **p < 0.01, ***p < 0.001 (versus control). ^#^*p* < 0.05, ^###^*p* < 0.001 (versus GDCA treated alone or GDCA with siNC).

Bile acids activate multiple G-protein-coupled receptors, especially S1PR2 [30]. Herein, we further explored whether S1PR2 mediated the GDCA-induced upregulation of ZBP1 expression. Firstly, RNA sequencing result showed that *S1PR2* mRNA level was much higher in BA patients than in control subjects (Fig. 7A). Similarly, qRT-PCR analysis also showed that mRNA level of *S1pr2* in liver was increased in BDL mice than in Sham mice (especially on the 14th day after BDL) (Fig. 7B). Secondly, we explored the expression pattern of *S1pr2* in different cell clusters through scRNA-seq analysis. The results showed that *S1pr2* was dominantly and highly expressed in macrophages, especially in BMDMs, rather than in other NPCs (Fig. 7C). More importantly, *S1pr2*^+^ BMDMs was mainly from BDL mouse model (Fig. 7C). Thirdly, S1PR2 expression in GDCA-treated BMDMs was examined. GDCA upregulated the mRNA expression of *S1pr2* in BMDMs in vitro (Fig. 7D). Thus, the above results indicated that S1PR2 played an important role in the regulation of ZBP1 expression. Then, BMDMs were incubated with the selective S1PR2 antagonist JTE-013, and the results showed that JTE-013 blocked the GDCA-induced upregulation of ZBP1 expression in mRNA and protein levels (Fig. 7E, G). Similarly, silencing *S1pr2* expression by siRNA also abrogated the GDCA-induced increase of ZBP1 expression (Supplementary Fig. 2A, and Fig. 7F, H). All these results confirmed that GDCA upregulated ZBP1 expression in mouse BMDMs through S1PR2.

### GDCA-induced upregulation of ZBP1 expression was a prerequisite for ZBP1/p-MLKL-mediated necroptosis in BMDMs

**Fig. 8:**
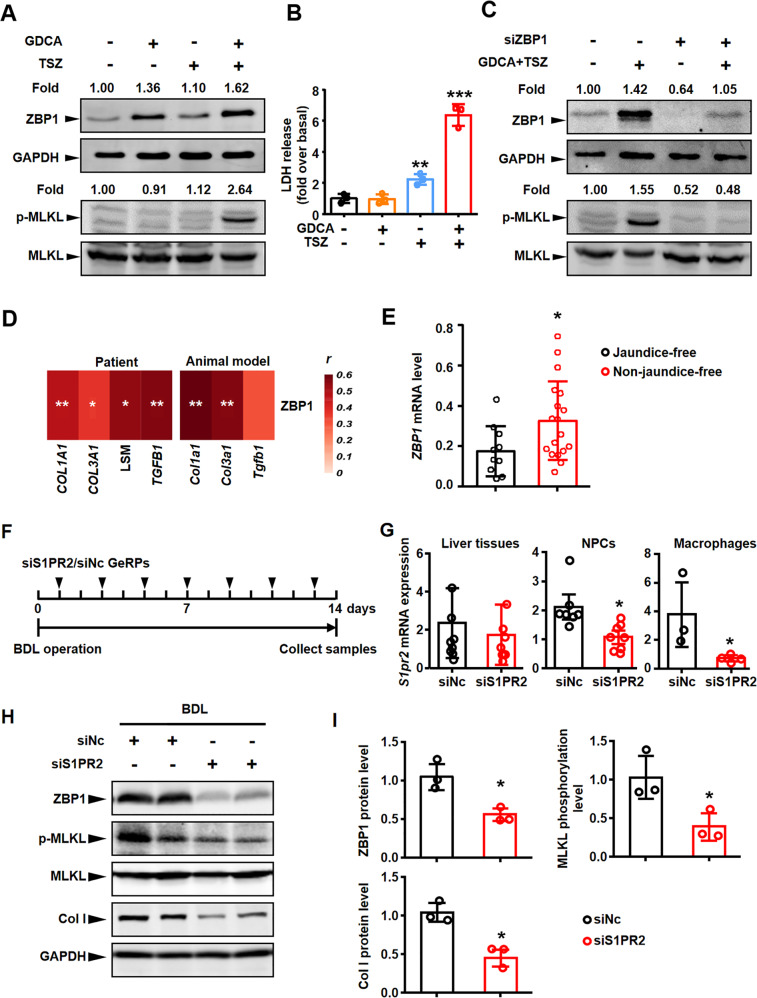
Blockage of macrophage S1PR2/ZBP1/p-MLKL alleviated necroptosis and further fibrosis in BDL liver. **A** Mouse BMDMs were treated with 100 μmol/L sodium glycodeoxycholate (GDCA) for 6 h, with or without treatment of TSZ (TNFα plus Smac mimetic and z-VAD-FMK) for 6 h. Western blot analysis for ZBP1 and p-MLKL in mouse BMDMs. Blots of ZBP1 were normalized to GAPDH, and blots of p-MLKL were normalized to MLKL. **B** Lactate dehydrogenase (LDH) activities in cell supernatant of mouse BMDMs were measured in GDCA-, TSZ- or GDCA + TSZ-treated BMDMs. **C** Effects of *Zbp1* siRNA on ZBP1 and p-MLKL protein expressions in mouse BMDMs treated with or without GDCA + TSZ. **D** The correlation between *ZBP1* mRNA level and LSM (liver stiffness measurement) or liver fibrosis marker gene levels in BA patient and mouse model livers. **E** The mRNA expression of *ZBP1* in BA liver tissues was compared between the jaundice-free and non-jaundice free groups after Kasai surgery at 6 months. **F** The schedule of mouse model. **G** mRNA expression of *S1pr2* in liver tissue, non-parenchymal cells (NPCs) and macrophages from BDL mouse livers treated with glucan-encapsulated NC or *S1pr2* siRNA particles (GeRPs). **H**, **I** Western blot analysis for ZBP1, p-MLKL, and Col I in the liver tissues from BDL mouse livers treated with NC (*n* = 3) or *S1pr2* (n = 3) siRNA-GeRPs. Bolts of p-MLKL were normalized to MLKL, blots of ZBP1 and Col I were normalized to GAPDH. Data are presented as the mean ± SEM. *p < 0.05, **p < 0.01, ***p < 0.001 (versus control).

We next explored whether GDCA induced necroptosis in BMDMs. BMDMs were incubated with GDCA to induce the increased expression of ZBP1 (Fig. 8A), and then treated with or without the classic necroptosis inducer TSZ (combination of TNFα, Smac mimetic, and a pan-caspase inhibitor z-VAD-FMK). The results showed that GDCA induced MLKL phosphorylation when combined with TSZ (Fig. 8A). However, GDCA or TSZ could not induce MLKL phosphorylation alone, respectively (Fig. 8A). Otherwise, we detected lactate dehydrogenase (LDH) release (one of necroptosis markers) to further confirm these results. GDCA combined with TSZ led to significant LDH release, whereas TSZ alone only led to a small amount of LDH release in BMDMs (Fig. 8B). In accordance with these results, RIPK1 and RIPK3 were also activated by GDCA and TSZ, not GDCA or TSZ alone (Supplementary Fig. 1C). Thus, these results confirmed that GDCA-induced upregulation of ZBP1 expression was a prerequisite for necroptosis in BMDMs.

To further determine whether ZBP1 mediated necroptosis triggered by GDCA and TSZ in BMDMs, we knocked down *Zbp1* expression in BMDMs using specific siRNA (Supplementary Fig. 2B). Here, we found that ZBP1 silencing blocked necroptosis induced by GDCA and TSZ (Fig. 8C, Supplementary Fig. 1D). These results demonstrated that GDCA-induced ZBP1 upregulation was an important basis of BMDM necroptosis.

### Selective knockdown of macrophage *S1pr2* decreased ZBP1 expression, attenuated necroptosis and fibrosis in BDL-injured livers

To further investigate the importance of ZBP1-mediated necroptosis in the progression of BA and BDL liver fibrosis, we next studied the relationship between ZBP1 expression and fibrosis marker expressions. The results of RNA sequencing and correlation analysis showed that *ZBP1* mRNA level was significantly correlated with fibrosis marker levels (*COL1A1, COL3A1,* and *TGFB1*) and liver stiffness measurement level (LSM, all *p* < 0.05, Fig. 8D). More importantly, the mRNA expression of *ZBP1* in liver tissues was correlated with jaundice clearance after Kasai surgery at six months (one of the important prognostic factors of BA), while *ZBP1* expression was significantly increased in the non-jaundice-free patients (Fig. 8E). Similarly, qRT-PCR results also showed that *Zbp1* mRNA level was significantly correlated with fibrosis markers (*Col1a1, Col3a1,* and *Tgfb1*) in BDL mouse livers (Fig. 8D). These results suggested that ZBP1 expression was correlated with liver fibrosis in BA and BDL.

Our previous study has already suggested that glucan-encapsulated siRNA particles (GeRPs) delivery system could selectively target macrophages and knock down gene expression in macrophages [21]. Then we injected mice intraperitoneally once every other day with *S1pr2*/negative control (NC) siRNA-GeRPs to selectively block S1PR2/ZBP1/p-MLKL axis in hepatic macrophages (Fig. 8F). The results of qRT-PCR showed that *S1pr2* mRNA expression was significantly decreased in macrophages isolated from livers. In liver NPCs, *S1pr2* expression decreased to a certain extent. However, in the liver tissues, the expression of *S1pr2* did not change significantly because the liver tissue was mainly composed of hepatocytes (Fig. 8G). These results further confirmed that siRNA-GeRPs could selectively target liver macrophages. Moreover, the selective blocking of macrophage *S1pr2* decreased ZBP1 and p-MLKL levels, indicating the reduction of necroptosis in BDL-injured livers (Fig. 8H, I). Western blot result also showed that the expression of collagen type I (Col I, encoding by *Col1a1*) was remarkably decreased in the BDL mouse livers treated with *S1pr2* siRNA-GeRPs (Fig. 8H, I). These results confirmed that the blockage of macrophage S1PR2/ZBP1/p-MLKL could alleviate necroptosis and further fibrosis in BDL liver, indicating the importance of GDCA/S1PR2/ZBP1/p-MLKL axis-mediated necroptosis in cholestatic liver fibrosis.

### Blockage of ZBP1/MLKL-mediated necroptosis attenuated BDL-induced liver injury/fibrosis

**Fig. 9:**
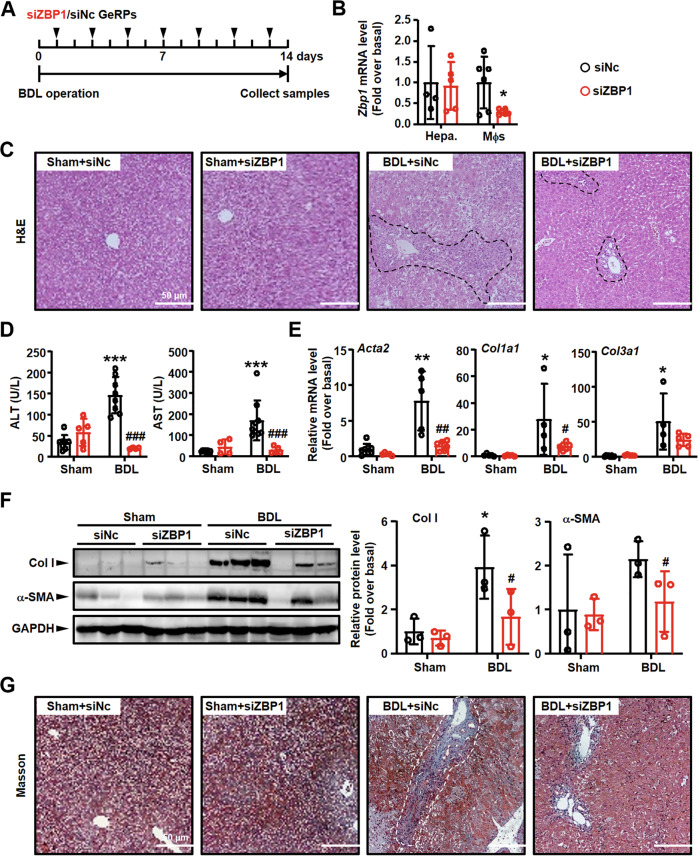
Specifically knockdown of macrophage *Zbp1* alleviated inflammation/fibrosis in BDL liver. **A** The schedule of mouse model. **B** mRNA expressions of *Zbp1* in hepatocytes (Hepa.) and macrophages (Mφs) from BDL mouse livers treated with NC or *Zbp1* siRNA-GeRPs. **C** Representative images of H&E staining. Black dashed: inflammation area. Scale bars: 50 μm. **D** Serum AST and ALT levels were detected in NC and *Zbp1* siRNA-GeRPs pretreated BDL livers. **E** mRNA expressions of fibrosis markers (*Acta2*, *Col1a1* and *Col3a1*) were detected by qRT-PCR. **F** Western blot analysis for Col I and α-SMA in the liver tissues from BDL mouse livers treated with NC and *Zbp1* siRNA-GeRPs. **G** Representative images of Masson staining. White dashed: collagen deposition area. Scale bars: 50 μm. Data are presented as the mean ± SEM. *p < 0.05, **p < 0.01, ***p < 0.001 (versus control). ^#^*p* < 0.05, ^##^*p* < 0.01, ^###^*p* < 0.001 (versus BDL alone).

We next employed *Zbp1* siRNA-GeRPs to further confirm the importance of ZBP1/p-MLKL-mediated necroptosis in liver fibrosis. Mice injected with *Zbp1* or NC siRNA-GeRPs were used to build BDL mouse model (Fig. 9A). First, the knockdown efficiency was studied. We isolated hepatocytes and macrophages from *Zbp1* or NC siRNA-GeRPs-injected mouse livers. *Zbp1* mRNA level was studied in these isolated cells. *Zbp1* siRNA-GeRPs markedly decreased *Zbp1* mRNA expression in macrophages, while that in hepatocytes was unchanged (Fig. 9B). Second, the degree of liver inflammation was analyzed by detecting immune cell infiltration. The results of H&E staining showed markedly immune cell infiltration in BDL livers, while *Zbp1* siRNA-GeRPs reduced inflammatory area in injured livers (Fig. 9C). Third, we examined serum AST/ALT to study the degree of liver injury. The results showed that BDL-induced increases of AST and ALT were significantly reduced by *Zbp1* siRNA-GeRPs (Fig. 9D). Fourth, the degree of liver fibrosis was also studied. The results of Masson staining showed the reduction of collagen deposition in *Zbp1* siRNA-GeRP-treated injured livers (Fig. 9G), which was in accordance with the decreases of *Acta2* (α-SMA, the marker of liver fibrosis), *Col1a1* (Col I) and *Col3a1* expressions in *Zbp1* siRNA-GeRPs-injection groups (Fig. 9E, F).

**Fig. 10:**
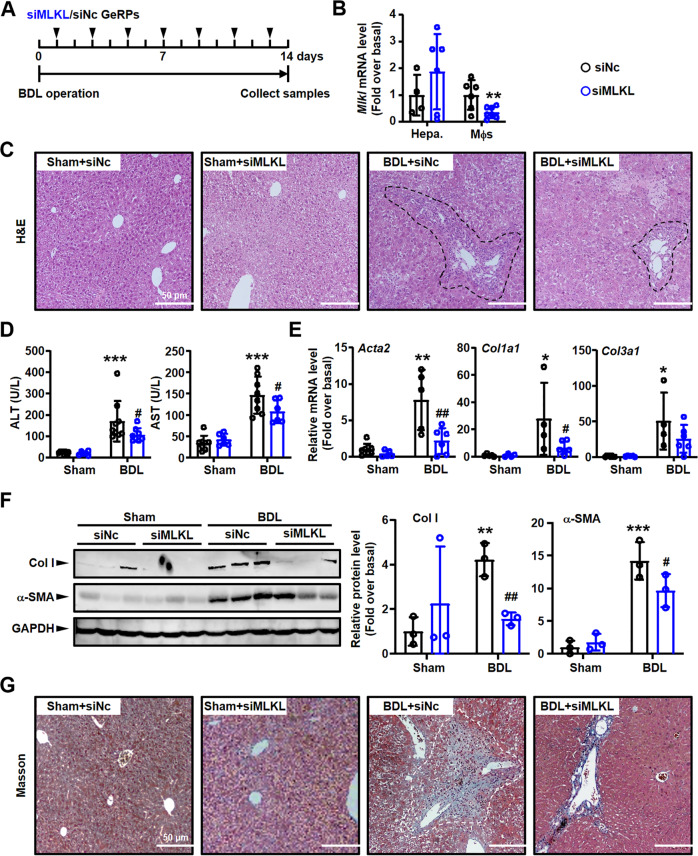
Specifically knockdown of macrophage *Mlkl* alleviated inflammation/fibrosis in BDL liver. **A** The schedule of mouse model. **B** mRNA expressions of *Mlkl* in hepatocytes (Hepa.) and macrophages (Mφs) from BDL mouse livers treated with NC or *Mlkl* siRNA-GeRPs. **C** Representative images of H&E staining. Black dashed: inflammation area. Scale bars: 50 μm. **D** Serum AST and ALT levels were detected in NC or *Mlkl* siRNA-GeRPs pretreated BDL livers. **E** mRNA expressions of fibrosis markers (*Acta2*, *Col1a1* and *Col3a1*) were detected by qRT-PCR. **F** Western blot analysis for Col I and α-SMA in the liver tissues from BDL mouse livers treated with NC or *Mlkl* siRNA-GeRPs. **G** Representative images of Masson staining. White dashed: collagen deposition area. Scale bars: 50 μm. Data are presented as the mean ± SEM. *p < 0.05, **p < 0.01, ***p < 0.001 (versus control). ^#^*p* < 0.05, ^##^*p* < 0.01 (versus BDL alone).

Similarly, the mice injected with *Mlkl* siRNA-GeRPs were also used to build BDL model (Fig. 10A). After isolating hepatocytes/macrophages from *Mlkl*/NC siRNA-GeRPs-injected livers to detect knockdown efficiency (Fig. 10B), the degree of liver injury/fibrosis were analyzed. In consistent with *Zbp1* siRNA-GeRP treatment group, *Mlkl* siRNA-GeRP injection also alleviated BDL-induced liver inflammation (Fig. 10C), injury (Fig. 10D) and fibrosis (Fig. 10E–G).

In conclusion, all these results showed that blockage of GDCA/S1PR2/ZBP1/p-MLKL axis-mediated necroptosis, which was an important player of cholestatic liver fibrosis, could be potential target of BA liver fibrosis.

## Discussion

BA, the most common cause of neonatal cholestasis, results from an fibroinflammatory obstruction of intrahepatic and extrahepatic biliary tree progressing to liver fibrosis and always requiring liver transplantation for survival [1, 31]. However, the limited understanding of the pathogenesis of this devastating disease hinders the development of novel medical therapies. Our study for the first time demonstrated the vital role of macrophage necroptosis mediated by ZBP1/p-MLKL to the pathology of BA liver fibrosis. We also confirmed that GDCA upregulated ZBP1 expression in mouse BMDMs through S1PR2, and the increased expression of ZBP1 was a prerequisite for ZBP1/p-MLKL-mediated necroptosis in BMDMs. More importantly, selective knockdown of *S1pr2*, *Zbp1* or *Mlkl* in macrophage decreased ZBP1/p-MLKL-mediated necroptosis, and further attenuated liver fibrosis in BDL-induced cholestatic liver injury. These results suggest that GDCA/S1PR2/ZBP1/p-MLKL-mediated necroptosis in macrophages is a key pathological feature in BA, and it plays vital role in the pathogenesis of BA liver fibrosis.

In previous studies, macrophages are proved as important players of liver injury. The increased CD11b^+^ monocytes/macrophages and CD68^+^ macrophages in BA liver are positively correlated with the progression of inflammation, fibrosis, and angiogenesis [19, 32]. Our previous studies have also shown that massive BMDMs engraft to the damaged liver, and display pro-inflammatory and pro-fibrotic effects in BDL mice [21, 33, 34]. Furthermore, recent studies confirm that p-MLKL-mediated macrophage necroptosis leads to the pathogenesis of respiratory syncytial virus infection [6], NASH [14], and inflammatory arthritis [35]. In this study, we observed that ZBP1/p-MLKL-mediated necroptosis occurred in macrophages of BA, and similarly in BDL mice. scRNA-seq results also confirmed that ZBP1^+^ BMDMs were existed in fibrotic liver and were involved in the cell death and inflammation pathways. These results suggest that, in addition to the traditional pro-inflammatory function, macrophages also play an important role in inflammatory and fibrotic diseases via necroptosis. Besides macrophages, necroptosis also occurs in other kinds of liver cells during liver fibrogenesis. For example, recent research has proved that *Mlkl* knockout significantly alleviates BDL-induced mouse liver fibrosis by reducing hepatocyte necroptosis and hepatic stellate cell activation [36]. All these results indicate that necroptosis is a vital player of cholestatic liver injury/fibrosis. However, our data showed that ~45% necroptotic cells were macrophages in injured livers, indicating the importance of macrophage necroptosis.

In injured liver, macrophages are composed by two major components, resident KCs and recruited BMDMs. To better distinguish these two kinds of macrophages, we employed chimera mouse model in which bone marrow cells were labeled with EGFP. It should be noticed that many published researches have proved that parts of KCs could be killed and replaced by bone marrow monocytes after lethal irradiation [33, 37, 38]. However, the recipient mice used in our experiments were undergone a four-week bone marrow rebuilding, during which period, the influences of the irradiation could be repaired [39, 40]. Herein, the effects of KC damage/depletion could be excluded in our study.

ZBP1 is an innate nucleic acid sensor that has important roles in regulating inflammatory diseases and host defense by orchestrating necroptosis and inflammation [17, 35, 41]. After being activated, ZBP1 plays key roles in the phosphorylation and activation of MLKL who further forms pores in the membrane to execute necroptosis [41, 42]. Beyond the previously known role in antiviral defense, recent studies have reported that ZBP1/p-MLKL-mediated necroptosis disrupts homeostasis of the epithelial barrier and promotes bowel inflammation [43], and plays a role in skin inflammation [44, 45]. In the current study, the levels of ZBP1 and p-MLKL were significantly upregulated in BA and BDL livers. Selective blockage of S1PR2/ZBP1/p-MLKL axis in macrophage attenuated necroptosis and liver fibrosis in BDL mice. Besides, we also employed necroptosis inhibitor targeting MLKL (Necrosulfonamide, NSA) to explore the role of necroptosis in liver fibrosis following recent publications [46, 47]. The results showed that NSA alleviated BDL-induced liver injury, inflammation, and fibrosis significantly (Supplementary Fig. 3). However, NSA has been reported as inhibitor of human MLKL, not murine MLKL [48]. Since NSA is also reported as inhibitor of GSDMD-mediated pyroptosis [49–51], which is also involved in liver injury/fibrosis [52], we consider that NSA is likely to attenuate BDL-induced liver fibrosis via inhibiting pyroptosis (maybe not necroptosis).

Former studies have indicated that the upregulation of ZBP1 expression is the basis of its role in regulating necroptosis [53–55]. Previous study has indicated that M1 but not M2 subtypes of macrophage is more susceptible to necroptosis under the stimulation of TSZ, which may be related to the upregulation of ZBP1 in M1 macrophages [53]. Other study also shows that the increased expression of ZBP1 in alveolar macrophages is the fundamental of lipopolysaccharide induced necroptosis in lung injury [54]. In the current study, we also proved that BMDM necroptosis was based on GDCA induced upregulation of ZBP1 expression, since GDCA combined with TSZ induced BMDM necroptosis while TSZ alone had little effect on it. This result was consistent with previous study, in which TSZ alone did not induce necroptosis in BMDMs [53]. In vivo, blockage of macrophage S1PR2 in BDL-injured livers could reduce ZBP1 expression, and further the level of p-MLKL. These findings suggested that the induction of ZBP1 was a prerequisite for the enhanced necroptosis, which further confirmed the importance of GDCA-induced ZBP1 expression in BMDM necroptosis. However, the specific mechanism of ZBP1 activation in macrophages in BA is still unclear, which requires further research.

TSZ is a kind of necroptosis inducer whose functions are dependent on RIPK1. Herein, we also detected p-RIPK1 level in BMDMs. The results proved that ZBP1 was an important player in TSZ-induced RIPK1 activation and necroptosis. The relationship between ZBP1 and RIPK1 is still a controversial and important issue to understand the mechanism underlying necroptosis. Research about skin inflammation report that RIPK1 inhibits ZBP1-induced RIPK3-MLKL activation and necroptosis, since RIPK1 prevents ZBP1 from binding and activating RIPK3 [44, 56]. However, there are also many researchers have reported that RIPK1 is an important downstream factor involving ZBP1-indcued necroptosis and inflammation [17, 41, 57, 58]. Our current results further support this conclusion.

Cholestasis is the important pathological feature of BA, which is characterized by the accumulation of hepatic biliary constituents, especially the conjugated bile acid [59, 60]. The conjugated bile acid levels in BA are significantly elevated in plasma and livers, including TCA, GCA, and GDCA [24–26]. Similarly, in the BDL mouse model, more than 99% of bile acids in liver and serum are conjugated bile acids [29]. In this study, conjugated bile acids GDCA induced the ZBP1 expression through S1PR2. However, high doses of GDCA failed to up-regulate ZBP1 expression in BMDMs. We consider that the decreased cell viability is responsible for the down-regulation of ZBP1 expression after treatment with high concentration of GDCA. In previous studies, the cytotoxic effects of high bile acid concentration have been reported. Fox example, deoxycholic acid (DCA, 200 μM and beyond) exhibits a dose-dependent inhibitory effect to cell proliferation [61]. Similarly, lithocholic acid significantly inhibits cell proliferation in a dose-dependent manner (60–180 μM) [62]. Treatment with 10–100 μM chenodeoxycholic acid and DCA also lead to concentration-dependent deleterious effects on cell viability under starvation [63]. On the other hand, the mechanism underlying GDCA-induced ZBP1 upregulation is still unclear. Former studies show that conjugated bile acids [TCA, GCA, GDCA, and taurodeoxycholic acid (TDCA)] activate ERK1/2 and AKT signaling pathways through S1PR2 in primary rodent hepatocytes [64, 65]. Furthermore, increased hepatic concentrations of TDCA and GDCA significantly contribute to liver fibrosis via p38MAPK and ERK1/2 signaling [66]. Herein, it still needs further study to explore whether these signaling pathways involved in GDCA/S1PR2-induced ZBP1 expression.

In conclusion, ZBP1/p-MLKL-dependent macrophage necroptosis is triggered in BA, and similarly BDL-induced mouse cholestatic liver injury. The increased mRNA expression of ZBP1 in BA livers is correlated with liver fibrosis and prognosis. Furthermore, the results of the current study demonstrate that conjugated bile acid GDCA upregulates ZBP1 expression in mouse BMDMs via S1PR2, and induces ZBP1/p-MLKL-dependent necroptosis on the basis of increased ZBP1 expression. Selective knockdown of macrophage *S1pr2*, *Zbp1* or *Mlkl* in BDL-injured livers blocks S1PR2/ZBP1/p-MLKL axis, and further attenuates necroptosis even fibrosis. Our results help in better understanding the role of GDCA/S1PR2/ZBP1/p-MLKL mediated macrophage necroptosis in cholestatic liver diseases, and might provide novel therapeutic strategies to attenuate BA liver fibrosis.

## Materials and methods

### Reagents

Sodium taurocholate hydrate (TCA, 86339), glycocholic acid hydrate (GCA, G2878), and sodium glycodeoxycholate (GDCA, G9910) were obtained from Sigma-Aldrich (St. Louis, MO, USA). JTE-013 (10009458) was obtained from Cayman Chemical (Ann Arbor, MI, USA). Necroptosis inducer kit TSZ (C1058S, TNFα plus Smac mimetic and a pan-caspase inhibitor z-VAD-FMK) was obtained from Beyotime (Shanghai, China). Necrosulfonamide (NSA, HY-100573) was obtained from MCE (NJ, USA).

### Human liver specimens

A total of 31 liver specimens were retrieved from BA patients undergoing Kasai surgery. Twenty normal adjacent non-tumor liver tissues taken from hepatoblastoma patients were used as healthy controls (HC). All patients’ guardians provided written informed consent. The patients’ characteristics are presented in Supplementary Table 1. This study was approved by the Medical Ethics Committee of the Beijing Children’s Hospital (2019-k-386).

### TEM analysis

Fresh liver biopsy specimens were immediately immersed in glutaraldehyde at 4 ˚C overnight, then fixed in 1% osmium tetroxide. After dehydration, samples were embedded in epoxy resin. After polymerization, several sections were placed onto copper grids and then stained with aqueous uranyl acetate and Reynold’s lead citrate. TEM assay was performed under a transmission electron microscope (JEM-1400, Jeol).

### Mouse model

Male ICR mice, 30.0 ± 3.0 g were used in this study. Mice received lethal irradiation (8 Grays) and immediately received transplantation by a tail vein injection of 1 × 10^6^ whole bone marrow cells obtained from 3-week-old EGFP transgenic mice. Four weeks later, mice of bone marrow-rebuild were subjected to BDL-induced liver injury. All mice were age-matched and randomized into the different groups. Sham-operated mice (sham, used as controls) underwent a laparotomy with exposure, but no ligation of the common bile duct was performed. Mice were anesthetized and sacrificed at 7 or 14 days after operation (*n* = 8 per group). All animal works were performed to the Ethics Committee of Capital Medical University and in accordance with the approved guidelines (approval number: AEEI-2014-131).

### Preparation and in vivo administration of GeRPs

The preparation of siRNA-GeRPs was performed as described in our former study [21]. Male ICR mice, 30.0 ± 3.0 g, at 6 weeks of age were anesthetized and were performed BDL or sham operation. The intraperitoneally injection of *S1pr2*, *Zbp1*, *Mlkl* or NC siRNA-GeRPs (1.5 mg GeRPs/kg of body weight) was performed one day after operation, then once every second day with the same dose for the following 12 days. Mice were anesthetized and sacrificed at 14 days after BDL or sham operation (*n* = 6 per group). The siRNA sequences were described as below.

### Isolation and culture of BMDMs

Primary mouse BMDMs were isolated from the tibia and femur bone marrow of ICR mice (20.0 ± 1.0 g). Macrophage colony-stimulating factor (M-CSF) was secreted by L929 cells and was used in the form of L929-conditioned medium. Bone marrow cells were grown in culture dishes for seven days in the presence of PRMI-1640 medium (Gibco Laboratories, Grand Island, NY) with 10% L929-contitioned medium. After 7 days, cells were serum starved for 6 h prior to treatment with TCA, GCA, GDCA, and TSZ.

### Mouse liver NPC and macrophage isolation

Livers were perfused with PBS twice and were minced on the ice. Then, collagenase type IV and DNase I were used to digest livers for 30 min at 37 °C with gentle shaking. Digested extracts were filtrated through 70 μm cell strainers to obtain single-cell suspensions. The cell suspension was subjected to density gradient (Histopaque-1077 and 1119, Sigma-Aldrich, St. Louis, MO, USA) centrifugation at 2000 rpm for 20 min. NPCs were collected from the upper layer of the interface after centrifugation and washed twice with PBS. Macrophages were separated using low-speed centrifugation and 70%/30% percoll density gradient centrifugation (middle layer).

### Immunofluorescence staining and TUNEL assay

Liver samples were fixed in 4% paraformaldehyde and embedded in Tissue Tek OCT compound. Frozen sections of 5 μm were used for immunofluorescence. Liver sections were blocked with 2% BSA, and then incubated with anti-ZBP1 (Adipogen, Zippy-1, AG-20B-0010, 1:100), anti-mouse MLKL (phospho S345, Abcam, ab196436, 1:50), anti-human MLKL (phospho S358, Abcam, ab187091, 1:50), CD68 (Santa Cruz, sc-9138, 1:100), CD86 (Santa Cruz, sc-28347, 1:100), CD11b (BD, 550282, 1:100), F4/80 (Santa Cruz, sc-59171, 1:100). FITC-conjugated affinipure goat anti-rabbit IgG, Cy3-conjugated affinipure goat anti-mouse, and Cy5-conjugated affinipure goat anti-rat IgG (1:200, Jackson Immunoresearch, PA, USA) were used as secondary antibodies. The sections of the livers were subjected to TUNEL analysis by using an FITC-TUNEL apoptosis detection kit (Yeasen, Shanghai, China) according to the manufacturer’s protocol. Finally, the sections were stained with DAPI and observed under a confocal microscope (Leica TCS SP8, Leica Microsystems, Mannheim, Germany).

### Western blot

Primary antibodies used in this study were as follows: anti-ZBP1 (Adipogen, Zippy-1, AG-20B-0010, 1:1000), anti-mouse MLKL (phospho S345, Abcam, ab196436, 1:1000), anti-human MLKL (phospho S358, Abcam, ab187091, 1:1000), anti-MLKL (Proteintech, 66675-1-Ig, 1:1000), anti-RIP (phospho S166, CST, 31122, 1:1000), anti-RIPK1 (CST, 3493, 1:1000), anti-RIPK3 (phosphor T231 + 232, Abcam, ab222320, 1:1000), anti-RIPK3 (Santa Cruz, sc-374639, 1:1000), COL1A1 (Abcam, Ab21286, 1:2000), α-SMA (Sigma Aldrich, A2547, 1:1000), and the appropriate IRDyeTM 800-conjugated secondary antibody (LI-COR, 926-32210, 926-32211, 1:10,000). Signals were detected using the Odyssey Imaging System (LI-COR Biosciences, Lincoln, NE, USA) and analyzed with Odyssey software. Results were normalized relative to GAPDH (CST, 2118 S, 1:1000) or α-tubulin (Proteintech, 11224-1-AP, 1:1000) expression.

### RNA extraction, sequencing, and analysis

Total RNAs were extracted from livers. The RNA libraries were constructed by poly(A) protocol. Fragmentation was performed using Fragmentation Buffer. The two strands of cDNA were synthesized using a random hexamer primer and M-MuLV Reverse Transcriptase (RNase H Minus) and DNA polymerase I and RNase H, respectively. The Illumina NovaSeq 6000 system was employed to sequence the cDNA libraries with paired-end 150 bp reads. The paired-end reads were aligned to human reference genome by STAR 2.7.9a [67] with Ensembl gene annotation (GRCh38/hg38 assembly). The mapped reads were then processed by Stringtie v2.1.4 to quantitate the gene expression against the Ensembl transcript annotation [68]. The count-based gene expression data was normalized and processed by R DESeq2 package [69].

### scRNA-seq and data analysis

scRNA-seq was performed by Capitalbio Technology Corporation (Beijing, China) as described [70]. GO analysis and pathway analysis were performed using Metascape [71]. Benjamini & Hochberg adjusted *p*-value <0.05 was recommended to present the significantly differential terms.

### qRT-PCR

Total RNA was extracted from cells or liver frozen specimens using GeneJET RNA Purification Kit (Thermo Scientific, Vilnius, Lithuania). The quantity and purity of RNA was determined by NanoDrop 2000 spectrophotometer (Thermo Fisher Scientific). cDNA was synthesized using oligo (dT) and M-MLV reverse transcriptase (Invitrogen). qRT-PCR was performed using SYBR Green qPCR Master Mix on the ABI 7300 TH Real-Time PCR System (Applied Biosystems, Foster City, CA, USA). All qRT-PCR assays and independent experiments were performed in triplicate respectively. Relative mRNA expression normalized to the house-keeping gene 18S rRNA was carried out using 2-ΔΔCt method. All primers were synthesized by Sangon Biotech (Shanghai, China). Primers used for qRT-PCR were as follows: 18S rRNA, sense, 5′-GTA ACC CGT TGA ACC CCA TT-3′, and anti-sense, 5′-CCA TCC AAT CGG TAG TAG CG-3′; mouse *Zbp1*, sense, 5′-CTC CTG CAA TCC CTG AGA ACT-3′, and anti-sense, 5′-GGC TAC ATG GCA AGA CTA TGT C-3′; mouse *S1pr2*, sense, 5′-TTC TGG AGG GTA ACA CAG TGG T-3′, and anti-sense, 5′-ACA CCC TTT GTA TCA AGT GGC A-3′; mouse *Col1a1*, sense, 5′-AGG GCG AGT GCT GTG CTT T-3′, and anti-sense, 5′-CCC TCG ACT CCT ACA TCT TCT GA-3′; mouse *Col3a1*, sense, 5′-TGA AAC CCC AGC AAA ACA AAA-3′, and anti-sense, 5′-TCA CTT GCA CTG GTT GAT AAG ATT AA-3′; mouse *Acta2*, sense, 5′-ATG CTC CCA GGG CTG TTT T-3′, and anti-sense, 5′-TTC CAA CCA TTA CTC CCT GAT GT-3′; human *ZBP1*, sense, 5′-GAC CTA GCC CAC CCT CTC AG-3′, and anti-sense, 5′-TTG TTC AAG GTG GCC TTC TCT-3′.

### RNA interference in vitro

siRNA targeting mouse *S1pr2* was from Invitrogen (Thermo Scientific, PA, USA). NC siRNA and siRNA targeting mouse *Zbp1* (GGG AAU GAC GAC AGC CAA A) or mouse *Mlkl* (GAA CCU GCC CGA UGA CAU U) were obtained from AuGCT (Beijing, China). Transient transfection of siRNA (40 nmol/L) was used by employing Lipofectamine RNAi MAX (Invitrogen, Carlsbad, CA) as recommended by the manufacturer. Cells were used to fulfill further experiments after 48 h.

### LDH activity assay

LDH release in cell supernatant were measured using LDH activity kit (Beyotime, C0017) according to the manufacturer’s protocol. Relative changes in the release of LDH were presented as fold ± SEM of individual control groups without treatment of necroptosis inducer TSZ and GDCA.

### Histology analysis

Liver tissues were fixed in 4% buffered formaldehyde. Liver tissue sections (5 μm) were stained with H&E staining for assess of injury, and Masson staining for extent of collagen deposition.

### Biochemical assays

Serum was separated and stored at −80 °C until use. Serum alanine aminotransferase (ALT) and aspartate aminotransferase (AST) were measured by using commercial assay kits (Stanbio, Boerne, USA).

### Statistical analysis

The statistical analysis of the results was performed using GraphPad Prism® 7.00 software (San Diego, USA). Results were expressed as mean ± SEM. Statistical significance was assessed by Student’s *t* test or one-way ANOVA when appropriate. Correlation coefficients were calculated by Pearson’s test. *p* < 0.05 was considered significant. All results were verified in at least three independent experiments.

## Supplementary information

aj-checklist

Supplementary Figure 1-3 Table 1

Full unedited blots

## Data Availability

The datasets generated during and/or analyzed during the current study are available from the corresponding authors under reasonable request.

## References

[1] Bezerra JA, Wells RG, Mack CL, Karpen SJ, Hoofnagle JH, Doo E (2018). Biliary Atresia: clinical and research challenges for the twenty-first century. Hepatology..

[2] Verkade HJ, Bezerra JA, Davenport M, Schreiber RA, Mieli-Vergani G, Hulscher JB (2016). Biliary atresia and other cholestatic childhood diseases: advances and future challenges. J Hepatol.

[3] Luo Z, Jegga AG, Bezerra JA (2018). Gene-disease associations identify a connectome with shared molecular pathways in human cholangiopathies. Hepatology..

[4] Xiao Y, Wang Y, Liu Y, Wang W, Tian X, Chen S (2021). A nonbile acid farnesoid X receptor agonist tropifexor potently inhibits cholestatic liver injury and fibrosis by modulating the gut-liver axis. Liver Int.

[5] Maelfait J, Liverpool L, Bridgeman A, Ragan KB, Upton JW, Rehwinkel J (2017). Sensing of viral and endogenous RNA by ZBP1/DAI induces necroptosis. Embo J.

[6] Santos LD, Antunes KH, Muraro SP, de Souza GF, Da SA, Felipe JS (2021). TNF-mediated alveolar macrophage necroptosis drives disease pathogenesis during respiratory syncytial virus infection. Eur Respir J.

[7] Kesavardhana S, Kuriakose T, Guy CS, Samir P, Malireddi R, Mishra A (2017). ZBP1/DAI ubiquitination and sensing of influenza vRNPs activate programmed cell death. J Exp Med.

[8] Newton K, Dixit VM, Kayagaki N (2021). Dying cells fan the flames of inflammation. Science..

[9] Zhou W, Yuan J (2014). Snapshot: Necroptosis. Cell..

[10] Schwabe RF, Luedde T (2018). Apoptosis and necroptosis in the liver: a matter of life and death. Nat Rev Gastro Hepat.

[11] Wang H, Sun L, Su L, Rizo J, Liu L, Wang LF (2014). Mixed lineage kinase domain-like protein MLKL causes necrotic membrane disruption upon phosphorylation by RIP3. Mol Cell.

[12] Gunther C, He GW, Kremer AE, Murphy JM, Petrie EJ, Amann K (2016). The pseudokinase MLKL mediates programmed hepatocellular necrosis independently of RIPK3 during hepatitis. J Clin Invest.

[13] Gautheron J, Vucur M, Reisinger F, Cardenas DV, Roderburg C, Koppe C (2014). A positive feedback loop between RIP3 and JNK controls non-alcoholic steatohepatitis. Embo Mol Med.

[14] Tao L, Yi Y, Chen Y, Zhang H, Orning P, Lien E (2021). RIP1 kinase activity promotes steatohepatitis through mediating cell death and inflammation in macrophages. Cell Death Differ.

[15] Afonso MB, Rodrigues PM, Mateus-Pinheiro M, Simao AL, Gaspar MM, Majdi A (2021). RIPK3 acts as a lipid metabolism regulator contributing to inflammation and carcinogenesis in non-alcoholic fatty liver disease. Gut..

[16] Bleriot C, Dupuis T, Jouvion G, Eberl G, Disson O, Lecuit M (2015). Liver-resident macrophage necroptosis orchestrates type 1 microbicidal inflammation and type-2-mediated tissue repair during bacterial infection. Immunity..

[17] Kesavardhana S, Kanneganti TD. ZBP1: A STARG٨TE to decode the biology of Z-nucleic acids in disease. J Exp Med. 2020;217:e20200885. 10.1084/jem.20200885 PMC7336316 32584411

[18] Lages CS, Simmons J, Maddox A, Jones K, Karns R, Sheridan R (2017). The dendritic cell-T helper 17-macrophage axis controls cholangiocyte injury and disease progression in murine and human biliary atresia. Hepatology..

[19] Tian X, Wang Y, Lu Y, Wang W, Du J, Chen S (2021). Conditional depletion of macrophages ameliorates cholestatic liver injury and fibrosis via lncRNA-H19. Cell Death Dis.

[20] Mohanty SK, Ivantes CA, Mourya R, Pacheco C, Bezerra JA (2010). Macrophages are targeted by rotavirus in experimental biliary atresia and induce neutrophil chemotaxis by Mip2/Cxcl2. Pediatr Res.

[21] Hou L, Yang L, Chang N, Zhao X, Zhou X, Dong C (2020). Macrophage sphingosine 1-phosphate receptor 2 blockade attenuates liver inflammation and fibrogenesis triggered by NLRP3 inflammasome. Front Immunol.

[22] Hou L, Zhang Z, Yang L, Chang N, Zhao X, Zhou X (2021). NLRP3 inflammasome priming and activation in cholestatic liver injury via the sphingosine 1-phosphate/S1P receptor 2/Galpha((12/13))/MAPK signaling pathway. J Mol Med.

[23] Yang L, Han Z, Tian L, Mai P, Zhang Y, Wang L (2015). Sphingosine 1-phosphate receptor 2 and 3 mediate bone marrow-derived monocyte/macrophage motility in cholestatic liver injury in mice. Sci Rep-Uk.

[24] Vanden BT, Linkermann A, Jouan-Lanhouet S, Walczak H, Vandenabeele P (2014). Regulated necrosis: the expanding network of non-apoptotic cell death pathways. Nat Rev Mol Cell Biol.

[25] Zhao D, Zhou K, Chen Y, Xie W, Zhang Y (2020). Development and validation of bile acid profile-based scoring system for identification of biliary atresia: a prospective study. Bmc Pediatr.

[26] Zhou K, Wang J, Xie G, Zhou Y, Yan W, Pan W (2015). Distinct plasma bile acid profiles of biliary atresia and neonatal hepatitis syndrome. J Proteome Res.

[27] Golden J, Zagory JA, Fenlon M, Goodhue CJ, Xiao Y, Fu X (2018). Liquid chromatography-mass spectroscopy in the diagnosis of biliary atresia in children with hyperbilirubinemia. J Surg Res.

[28] Johansson H, Svensson JF, Almstrom M, Van Hul N, Rudling M, Angelin B (2020). Regulation of bile acid metabolism in biliary atresia: reduction of FGF19 by Kasai portoenterostomy and possible relation to early outcome. J Intern Med.

[29] Wang Y, Aoki H, Yang J, Peng K, Liu R, Li X (2017). The role of sphingosine 1-phosphate receptor 2 in bile-acid-induced cholangiocyte proliferation and cholestasis-induced liver injury in mice. Hepatology..

[30] Studer E, Zhou X, Zhao R, Wang Y, Takabe K, Nagahashi M (2012). Conjugated bile acids activate the sphingosine-1-phosphate receptor 2 in primary rodent hepatocytes. Hepatology..

[31] Mohanty SK, Donnelly B, Temple H, Ortiz-Perez A, Mowery S, Lobeck I (2021). High mobility group box 1 release by cholangiocytes governs biliary atresia pathogenesis and correlates with increases in afflicted infants. Hepatology..

[32] Taylor SA, Chen SY, Gadhvi G, Feng L, Gromer KD, Abdala-Valencia H (2021). Transcriptional profiling of pediatric cholestatic livers identifies three distinct macrophage populations. PLoS ONE.

[33] Han Z, Zhu T, Liu X, Li C, Yue S, Liu X (2012). 15-deoxy-Delta12,14 -prostaglandin J2 reduces recruitment of bone marrow-derived monocyte/macrophages in chronic liver injury in mice. Hepatology..

[34] Yang L, Yang L, Dong C, Li L (2018). The class D scavenger receptor CD68 contributes to mouse chronic liver injury. Immunol Res.

[35] Polykratis A, Martens A, Eren RO, Shirasaki Y, Yamagishi M, Yamaguchi Y (2019). A20 prevents inflammasome-dependent arthritis by inhibiting macrophage necroptosis through its ZnF7 ubiquitin-binding domain. Nat Cell Biol.

[36] Guo R, Jia X, Ding Z, Wang G, Jiang M, Li B (2022). Loss of MLKL ameliorates liver fibrosis by inhibiting hepatocyte necroptosis and hepatic stellate cell activation. Theranostics..

[37] Mai P, Yang L, Tian L, Wang L, Jia S, Zhang Y (2015). Endocannabinoid system contributes to liver injury and inflammation by activation of bone marrow-derived monocytes/macrophages in a CB1-dependent manner. J Immunol.

[38] Tian L, Li W, Yang L, Chang N, Fan X, Ji X (2017). Cannabinoid receptor 1 participates in liver inflammation by promoting M1 macrophage polarization via RhoA/NF-kappaB p65 and ERK1/2 pathways, respectively, in mouse liver fibrogenesis. Front Immunol.

[39] Russo FP, Alison MR, Bigger BW, Amofah E, Florou A, Amin F (2006). The bone marrow functionally contributes to liver fibrosis. Gastroenterology..

[40] Durum SK, Gengozian N (1978). The comparative radiosensitivity of T and B lymphocytes. Int J Radiat Biol Relat Stud Phys Chem Med.

[41] Kuriakose T, Kanneganti TD (2018). ZBP1: innate sensor regulating cell death and inflammation. Trends Immunol.

[42] Malireddi R, Kesavardhana S, Kanneganti TD (2019). ZBP1 and TAK1: master regulators of NLRP3 inflammasome/pyroptosis, apoptosis, and necroptosis (Pan-Optosis). Front Cell Infect Mi.

[43] Wang R, Li H, Wu J, Cai ZY, Li B, Ni H (2020). Gut stem cell necroptosis by genome instability triggers bowel inflammation. Nature..

[44] Lin J, Kumari S, Kim C, Van TM, Wachsmuth L, Polykratis A (2016). RIPK1 counteracts ZBP1-mediated necroptosis to inhibit inflammation. Nature..

[45] Jiao H, Wachsmuth L, Kumari S, Schwarzer R, Lin J, Eren RO (2020). Z-nucleic-acid sensing triggers ZBP1-dependent necroptosis and inflammation. Nature..

[46] Duan X, Liu X, Liu N, Huang Y, Jin Z, Zhang S (2020). Inhibition of keratinocyte necroptosis mediated by RIPK1/RIPK3/MLKL provides a protective effect against psoriatic inflammation. Cell Death Dis.

[47] Jiao J, Wang Y, Ren P, Sun S, Wu M (2019). Necrosulfonamide ameliorates neurological impairment in spinal cord injury by improving antioxidative capacity. Front Pharmacol.

[48] Liu S, Liu H, Johnston A, Hanna-Addams S, Reynoso E, Xiang Y (2017). MLKL forms disulfide bond-dependent amyloid-like polymers to induce necroptosis. Proc Natl Acad Sci USA.

[49] Rashidi M, Simpson DS, Hempel A, Frank D, Petrie E, Vince A (2019). The pyroptotic cell death effector gasdermin D is activated by gout-associated uric acid crystals but is dispensable for cell death and IL-1beta release. J Immunol.

[50] Wu YL, Ou WJ, Zhong M, Lin S, Zhu YY (2022). Gasdermin D inhibitor necrosulfonamide alleviates lipopolysaccharide/D-galactosamine-induced acute liver failure in mice. J Clin Transl Hepatol.

[51] Han C, Yang Y, Guan Q, Zhang X, Shen H, Sheng Y (2020). New mechanism of nerve injury in Alzheimer’s disease: beta-amyloid-induced neuronal pyroptosis. J Cell Mol Med.

[52] Gan C, Cai Q, Tang C, Gao J (2022). Inflammasomes and pyroptosis of liver cells in liver fibrosis. Front Immunol.

[53] Hao Q, Kundu S, Kleam J, Zhao ZJ, Idell S, Tang H (2021). Enhanced RIPK3 kinase activity-dependent lytic cell death in M1 but not M2 macrophages. Mol Immunol.

[54] Du XK, Ge WY, Jing R, Pan LH (2019). Necroptosis in pulmonary macrophages mediates lipopolysaccharide-induced lung inflammatory injury by activating ZBP-1. Int Immunopharmacol.

[55] Yuan F, Cai J, Wu J, Tang Y, Zhao K, Liang F (2022). Z-DNA binding protein 1 promotes heatstroke-induced cell death. Science..

[56] Udawatte DJ, Rothman AL (2021). Viral suppression of RIPK1-mediated signaling. Mbio..

[57] Muendlein HI, Connolly WM, Magri Z, Smirnova I, Ilyukha V, Gautam A (2021). ZBP1 promotes LPS-induced cell death and IL-1beta release via RHIM-mediated interactions with RIPK1. Nat Commun.

[58] Peng R, Wang CK, Wang-Kan X, Idorn M, Kjaer M, Zhou FY (2022). Human ZBP1 induces cell death-independent inflammatory signaling via RIPK3 and RIPK1. Embo Rep.

[59] Amaral JD, Viana RJ, Ramalho RM, Steer CJ, Rodrigues CM (2009). Bile acids: regulation of apoptosis by ursodeoxycholic acid. J Lipid Res.

[60] Karpen SJ, Kelly D, Mack C, Stein P (2020). Ileal bile acid transporter inhibition as an anticholestatic therapeutic target in biliary atresia and other cholestatic disorders. Hepatol Int.

[61] Li J, Zhang C, Li L, Hu X, Jia Y, Huang Y (2022). Folate deficiency enhances the in vitro genotoxicity of bile acids in human colon and liver cells. Mutagenesis..

[62] Feng Z, Jia C, Lin X, Hao H, Li S, Li F (2022). The inhibition of enterocyte proliferation by lithocholic acid exacerbates necrotizing enterocolitis through downregulating the Wnt/beta-catenin signalling pathway. Cell Proliferat.

[63] Yang X, Zhou Y, Li H, Song F, Li J, Zhang Y (2022). Autophagic flux inhibition, apoptosis, and mitochondrial dysfunction in bile acids-induced impairment of human placental trophoblast. J Cell Physiol.

[64] Nagahashi M, Takabe K, Liu R, Peng K, Wang X, Wang Y (2015). Conjugated bile acid-activated S1P receptor 2 is a key regulator of sphingosine kinase 2 and hepatic gene expression. Hepatology..

[65] Dent P, Fang Y, Gupta S, Studer E, Mitchell C, Spiegel S (2005). Conjugated bile acids promote ERK1/2 and AKT activation via a pertussis toxin-sensitive mechanism in murine and human hepatocytes. Hepatology..

[66] Xie G, Jiang R, Wang X, Liu P, Zhao A, Wu Y (2021). Conjugated secondary 12alpha-hydroxylated bile acids promote liver fibrogenesis. Ebiomedicine..

[67] Dobin A, Davis CA, Schlesinger F, Drenkow J, Zaleski C, Jha S (2013). STAR: ultrafast universal RNA-seq aligner. Bioinformatics..

[68] Pertea M, Pertea GM, Antonescu CM, Chang TC, Mendell JT, Salzberg SL (2015). StringTie enables improved reconstruction of a transcriptome from RNA-seq reads. Nat Biotechnol.

[69] Love MI, Huber W, Anders S (2014). Moderated estimation of fold change and dispersion for RNA-seq data with DESeq2. Genome Biol.

[70] Chang N, Tian L, Ji X, Zhou X, Hou L, Zhao X (2019). Single-cell transcriptomes reveal characteristic features of mouse hepatocytes with liver cholestatic injury. Cells-Basel.

[71] Zhou Y, Zhou B, Pache L, Chang M, Khodabakhshi AH, Tanaseichuk O (2019). Metascape provides a biologist-oriented resource for the analysis of systems-level datasets. Nat Commun.

