# Supplementary material for: Necroptosis of macrophage is a key pathological feature in biliary atresia via GDCA/S1PR2/ZBP1/p-MLKL axis
Source: Cell Death Dis. 2023 Mar 1;14(3):175. doi: 10.1038/s41419-023-05615-4 (PMC9977961; doi:10.1038/s41419-023-05615-4)
Supplement: Supplementary file 2 — Supplementary Figure 1-3 Table 1 [file 41419_2023_5615_MOESM2_ESM.pdf]

Necroptosis of macrophage is a key pathological feature in biliary atresia via GDCA/S1PR2/ZBP1/p-MLKL axis

Shen Yang[1,2]#, Na Chang[1]#, Weiyang Li[1]#, Ting Yang[2], Renmin Xue[1], Jing Liu[1], Li Zhang[3], Xingfeng Yao[4], Yajun Chen[5], Huanmin Wang[6], Lin Yang[1], Jinshi Huang[2]*, Liying Li[1]*

Supplementary Table 1. The comparison of clinical characteristics between biliary atresia and control groups.

Clinical characteristics	Biliary atresia (n = 31)	Control (n = 20)	*p* value
Boys (n, %)	16 (51.61)	12 (60.00)	0.557
Age (months, median, IQR)	2 (1, 3)	21 (12, 33)	< 0.001
Aspartate aminotransferase (U/L, median, IQR)	303.50 (215.70, 371.70)	85.50 (48.90, 722.20)	< 0.001
Alanine aminotransferase (U/L, median, IQR)	207.30 (136.00, 267.40)	39.10 (20.50, 263.50)	< 0.001
Total bilirubin (μmol/L, median, IQR)	168.70 (140.23, 203.84)	8.04 (6.43, 13.55)	< 0.001
Direct bilirubin (μmol/L, median, IQR)	125.75 (92.11, 147.72)	2.62 (0.79, 4.46)	< 0.001
Indirect bilirubin (μmol/L, median, IQR)	52.16 (44.22, 87.40)	7.15 (5.75, 12.26)	< 0.001
Total bile acid (μmol/L, median, IQR)	138.54 (102.22, 214.20)	6.33 (3.52, 7.64)	< 0.001
γ-GT (U/L, median, IQR)	786.40 (504.00, 1517.10)	42.60 (32.70, 93.60)	< 0.001

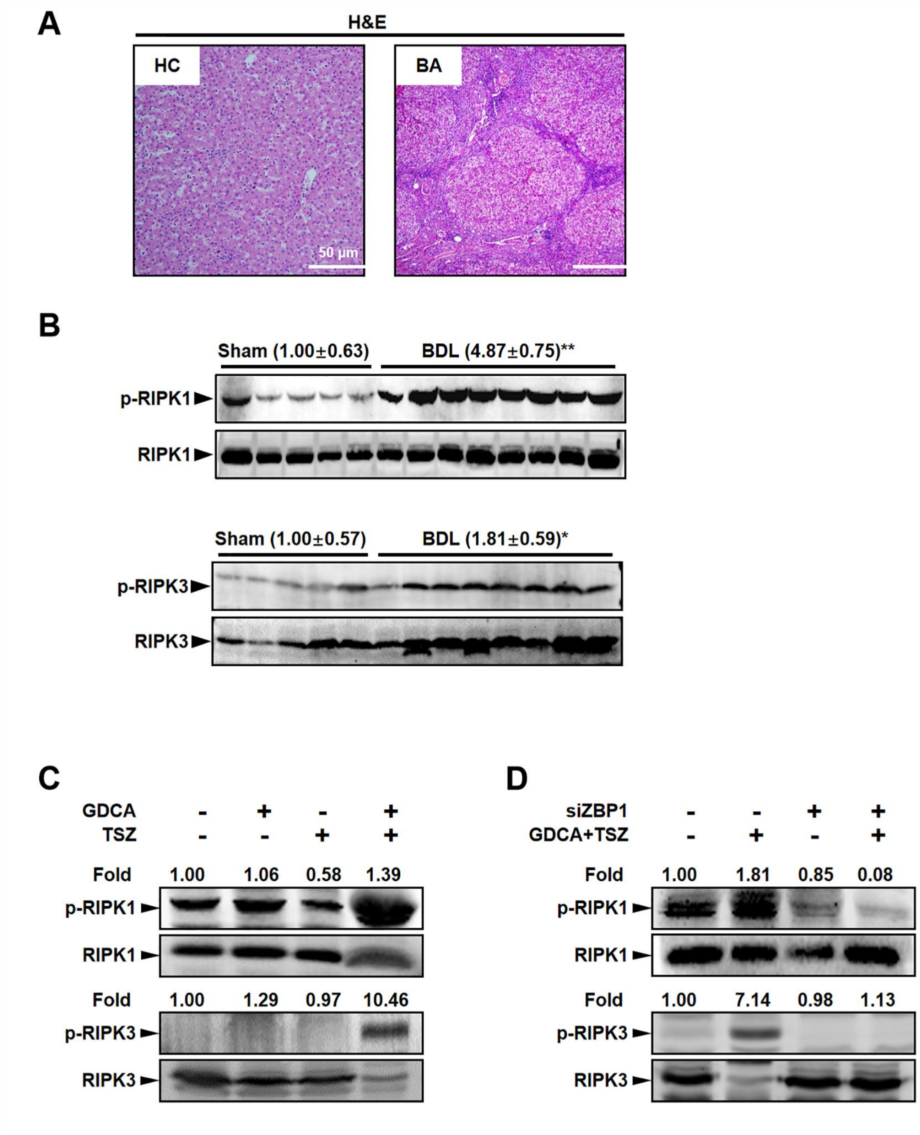

Supplementary Figure 1. The Pathological changes of BA livers and RIPK1/RIPK3 expressions in BDL or BMDMs.

A. Representative images of H&E staining in normal adjacent non-tumor livers (HC) and biliary atresia (BA) livers. Scale bar: 50 μm. **B.** Western blot analysis for p-RIPK1 and p-RIPK3 in the liver tissues from BDL (n=8) and Sham mice (n=5). **C.** Mouse BMDMs were treated with 100 μmol/L sodium glycodeoxycholate (GDCA) for 6 hours, with or without treatment of TSZ (TNFα plus Smac mimetic and a pan-caspase inhibitor z-VAD-FMK) for 6 hours. Western blot analysis for p-RIPK1 and p-RIPK3 in mouse BMDMs. **D.** Effects of *Zbp1* siRNA on p-RIPK1 and p-RIPK3 protein expression in mouse BMDMs. Bolts of p-RIPK1 or p-RIPK3 were normalized to RIPK1 or RIPK3, respectively. Data are presented as the mean ± SEM. * $p<0.05$, ** $p<0.01$ (versus control).

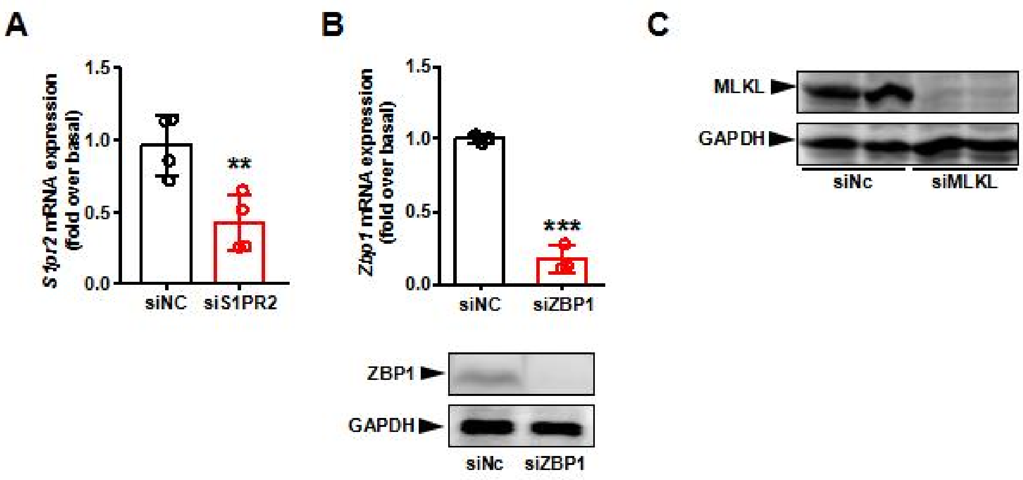

Supplementary Figure 2. The efficiency of siRNAs in mouse BMDMs

A-C. The efficiency of *S1pr2* (**A**), *Zbp1* (**B**) or MLKL (**C**) knockdown by the siRNA in mouse BMDMs. ** p<0.01, *** p<0.001 (versus control).

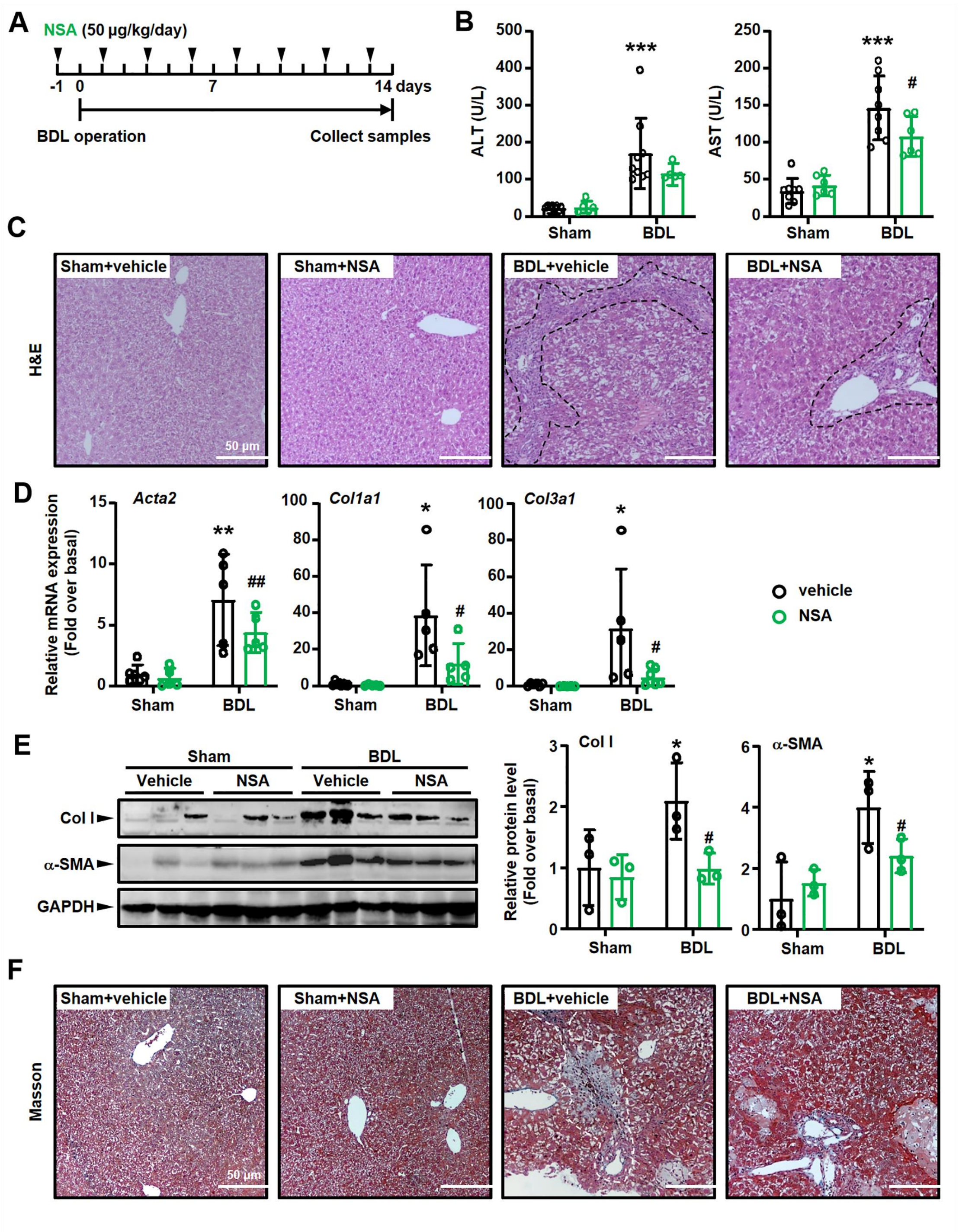

Supplementary Figure 3. **Necroptosis inhibitor (Necrosulfonamide, NSA) alleviates inflammation /fibrosis in BDL liver.**

A. The schedule of mouse model. **B**. Serum AST and ALT levels were detected in BDL livers with or without NSA treatment. **C**. Representative images of H&E staining. Black dashed: inflammation area. Scale bars: 50 µm. **D**. mRNA expressions of fibrosis markers (*Acta2*, *Col1a1* and *Col3a1*) were detected by qRT-PCR. **E.** Western blot analysis for Col I and α-SMA in the liver tissues from BDL mouse livers treated with or without NSA. **F**. Representative images of Masson staining. White

dashed: collagen deposition area. Scale bars: 50 μm. Data are presented as the mean ± SEM. * $p<0.05$, ** $p<0.01$, *** $p<0.001$ (versus control). # $p<0.05$, ## $p<0.01$ (versus BDL alone).
