# Supplementary material for: Necroptosis of macrophage is a key pathological feature in biliary atresia via GDCA/S1PR2/ZBP1/p-MLKL axis
Source: Cell Death Dis. 2023 Mar 1;14(3):175. doi: 10.1038/s41419-023-05615-4 (PMC9977961; doi:10.1038/s41419-023-05615-4)
Supplement: Supplementary file 3 — Full unedited blots [file 41419_2023_5615_MOESM3_ESM.pdf]

Fig. 1C

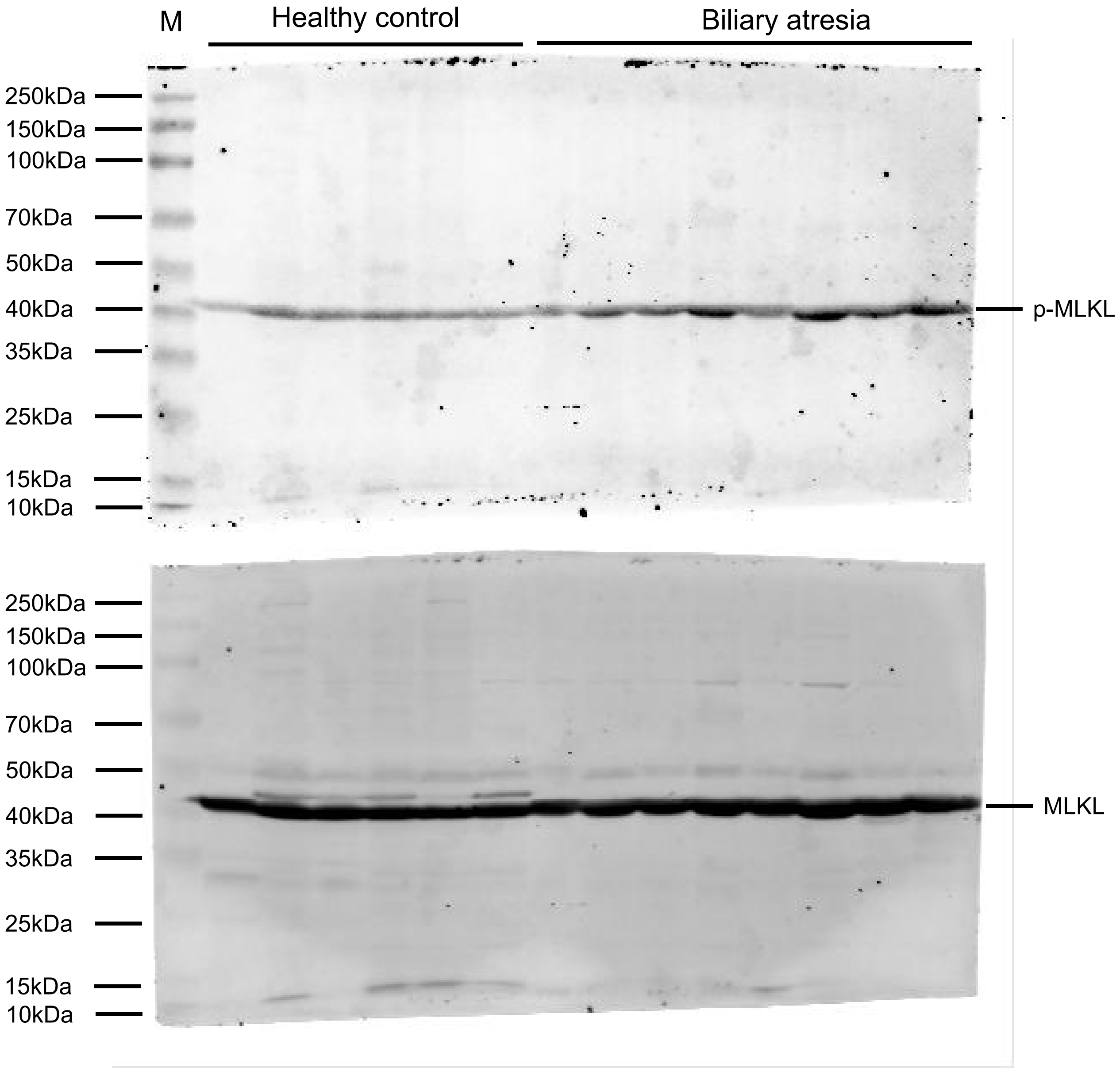
M
Healthy control
Biliary atresia
250kDa
150kDa
100kDa
70kDa
50kDa
40kDa
35kDa
25kDa
15kDa
10kDa
p-MLKL
250kDa
150kDa
100kDa
70kDa
50kDa
40kDa
35kDa
25kDa
15kDa
10kDa
MLKL

Fig. 1C

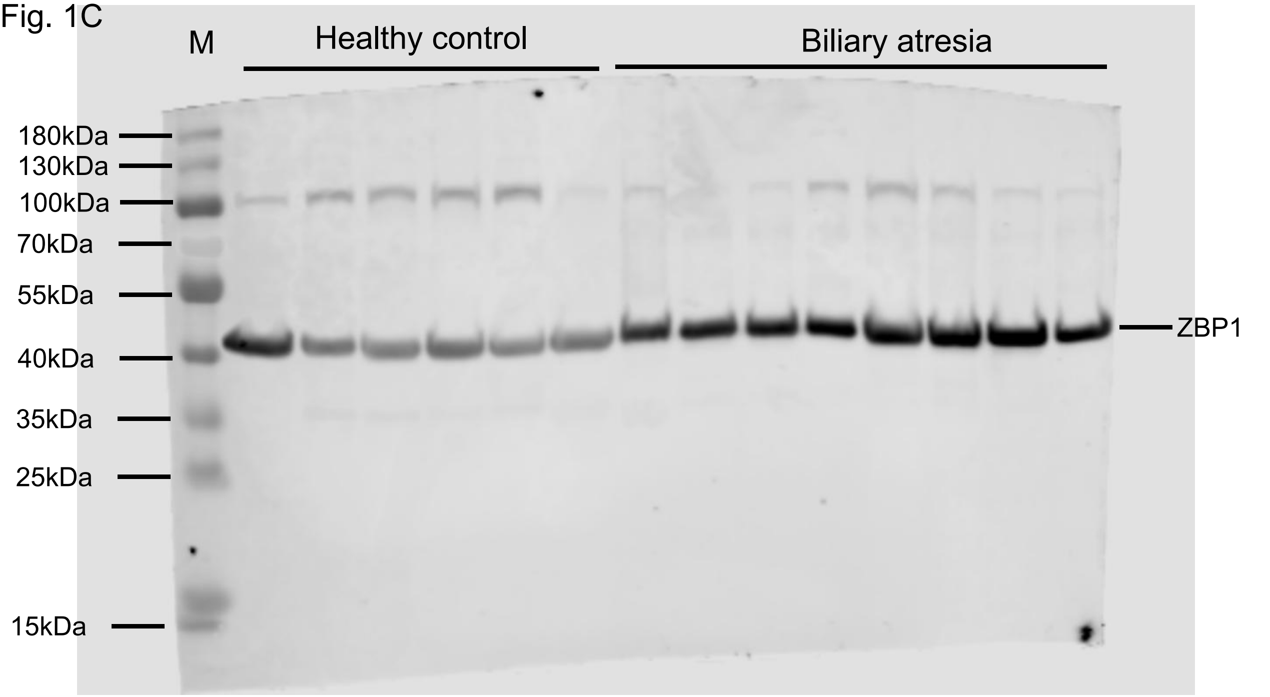

M
Healthy control
Biliary atresia
180kDa
130kDa
100kDa
70kDa
55kDa
40kDa
35kDa
25kDa
15kDa
ZBP1

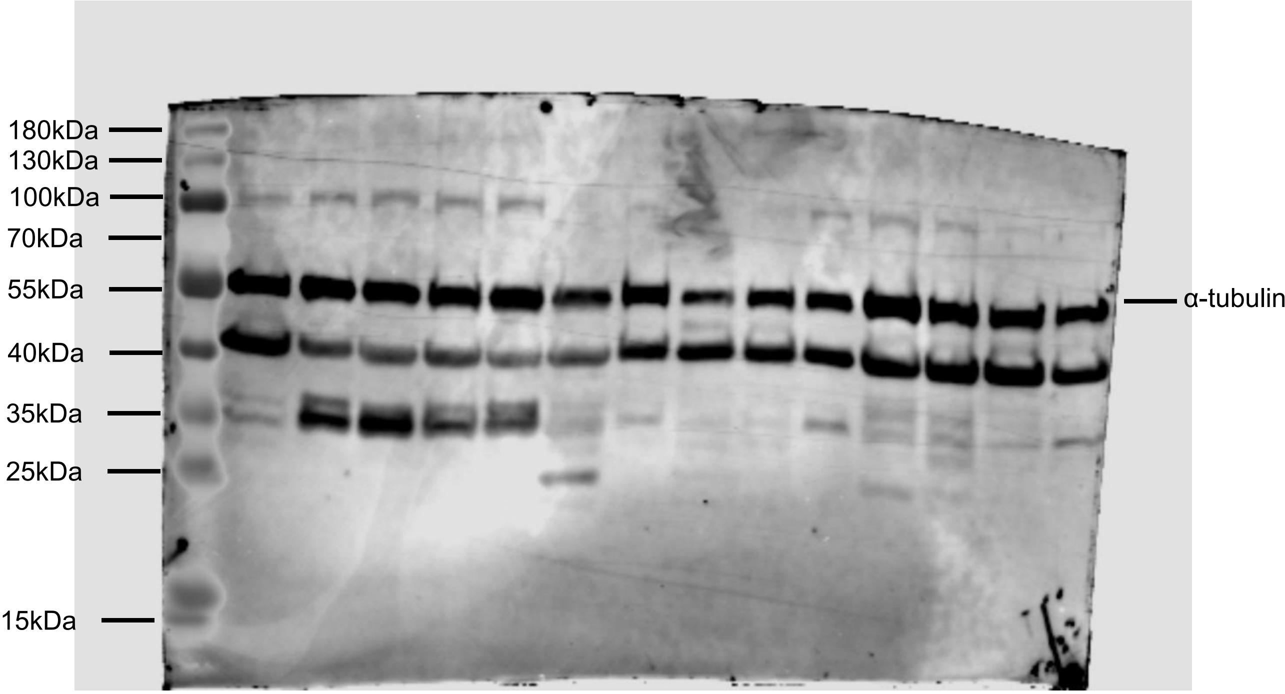

180kDa
130kDa
100kDa
70kDa
55kDa
40kDa
35kDa
25kDa
15kDa
α-tubulin

Fig. 3C

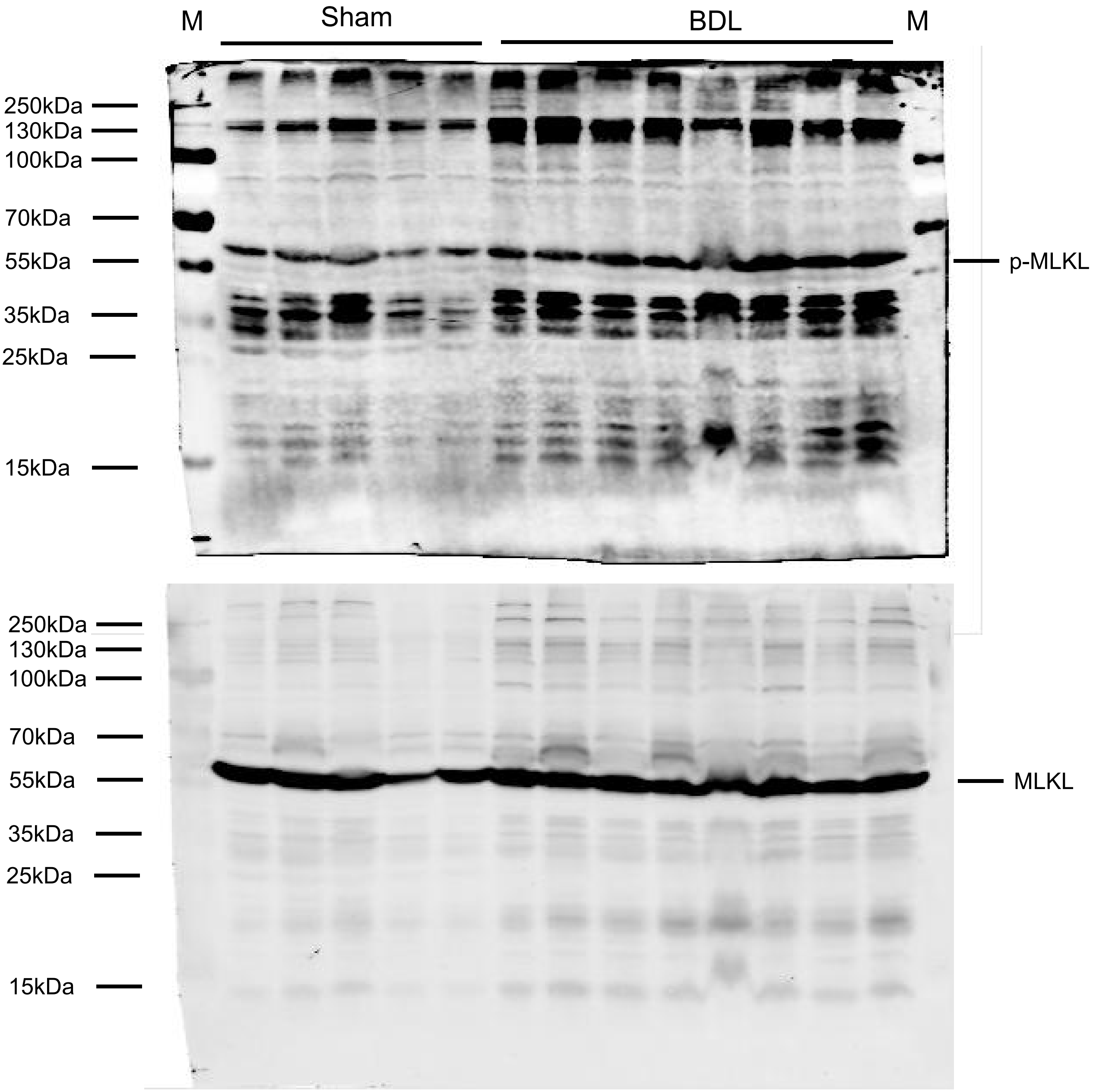
M
Sham
BDL
M
250kDa
130kDa
100kDa
70kDa
55kDa
35kDa
25kDa
15kDa
p-MLKL
250kDa
130kDa
100kDa
70kDa
55kDa
35kDa
25kDa
15kDa
MLKL

Fig. 3C

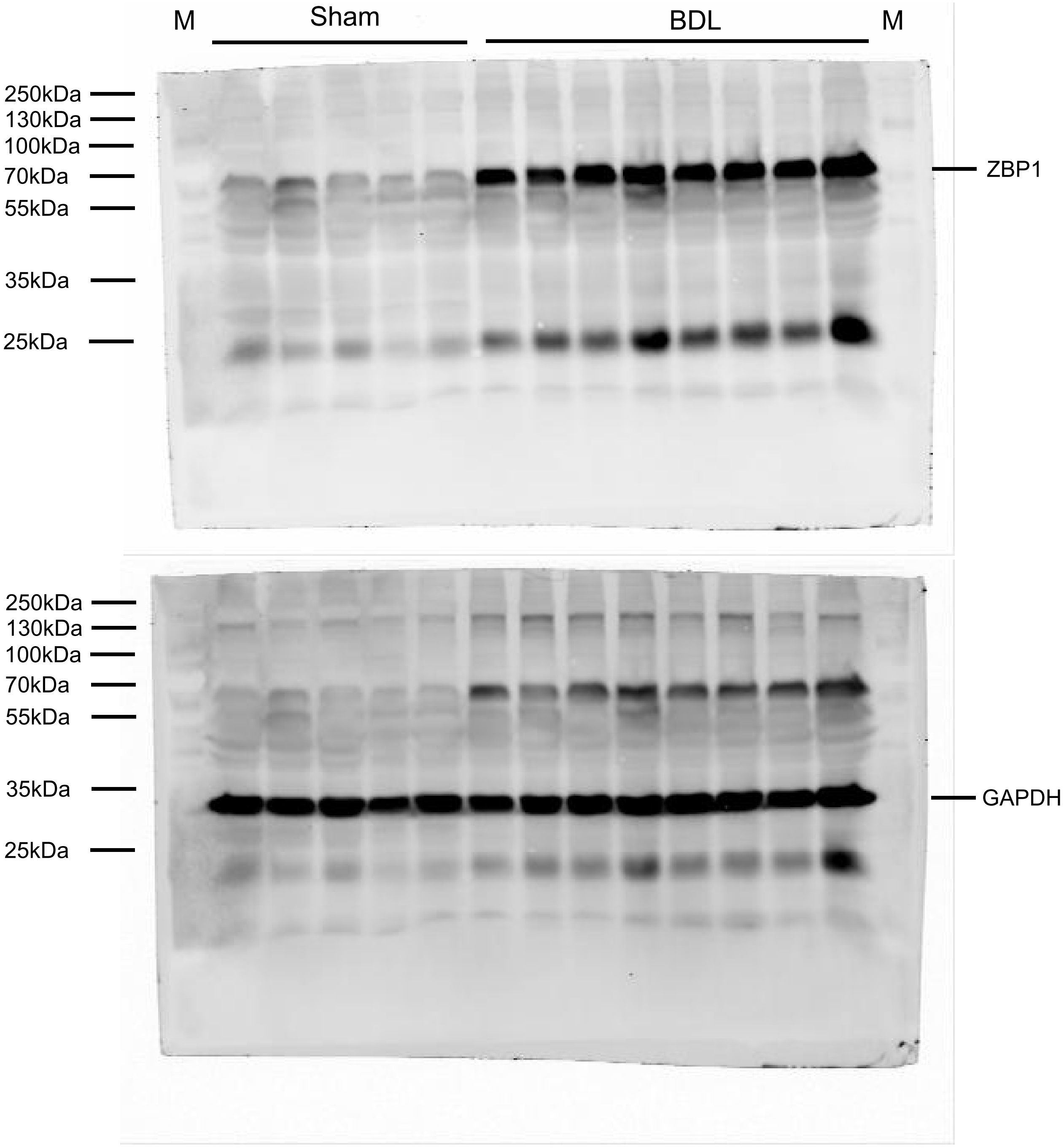
M
Sham
BDL
M
250kDa
130kDa
100kDa
70kDa
55kDa
35kDa
25kDa
ZBP1
250kDa
130kDa
100kDa
70kDa
55kDa
35kDa
25kDa
GAPDH

Fig. 6B

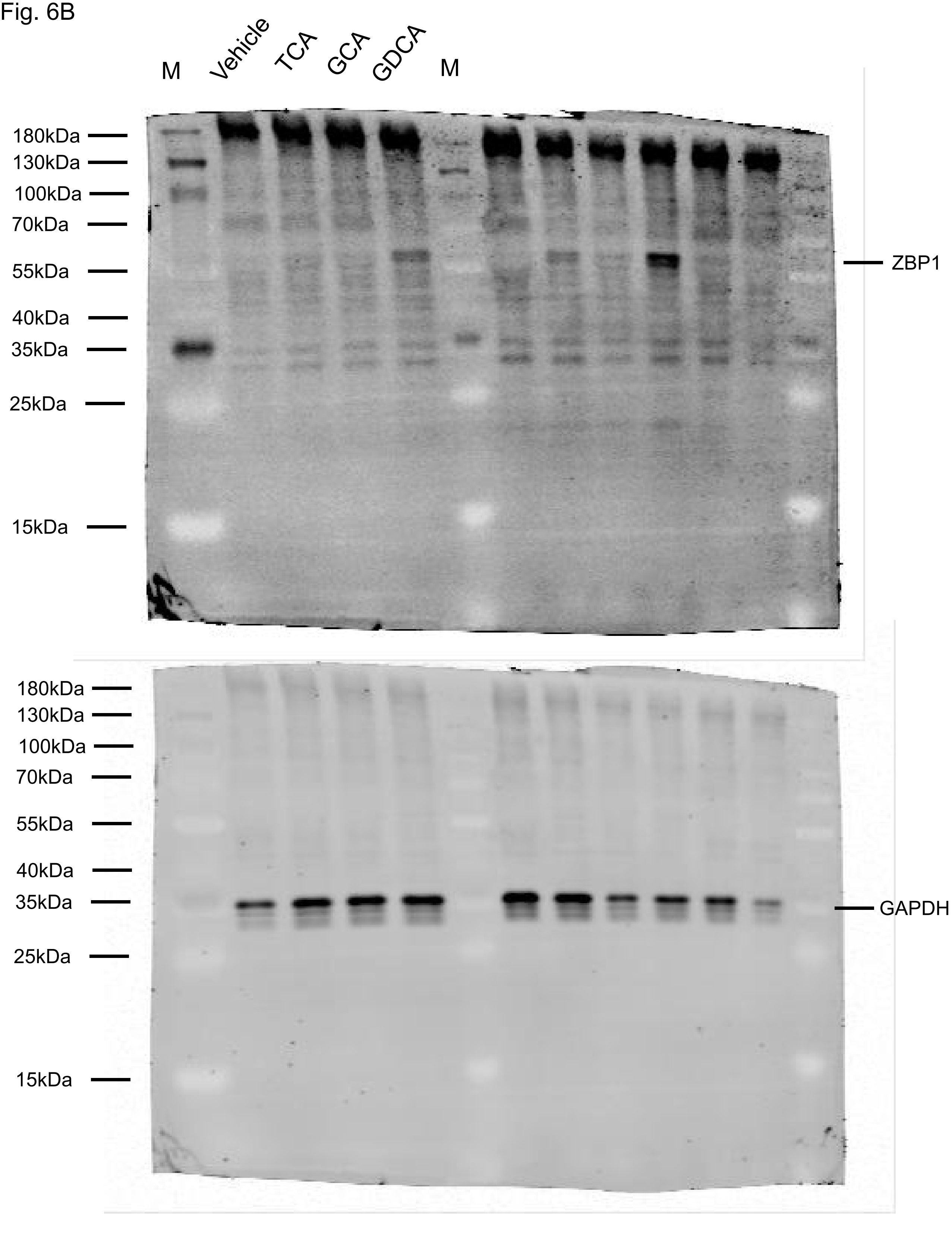
M
Vehicle
TCA
GCA
GDCA
M
180kDa
130kDa
100kDa
70kDa
55kDa
40kDa
35kDa
25kDa
15kDa
ZBP1
180kDa
130kDa
100kDa
70kDa
55kDa
40kDa
35kDa
25kDa
15kDa
GAPDH

Fig. 6E

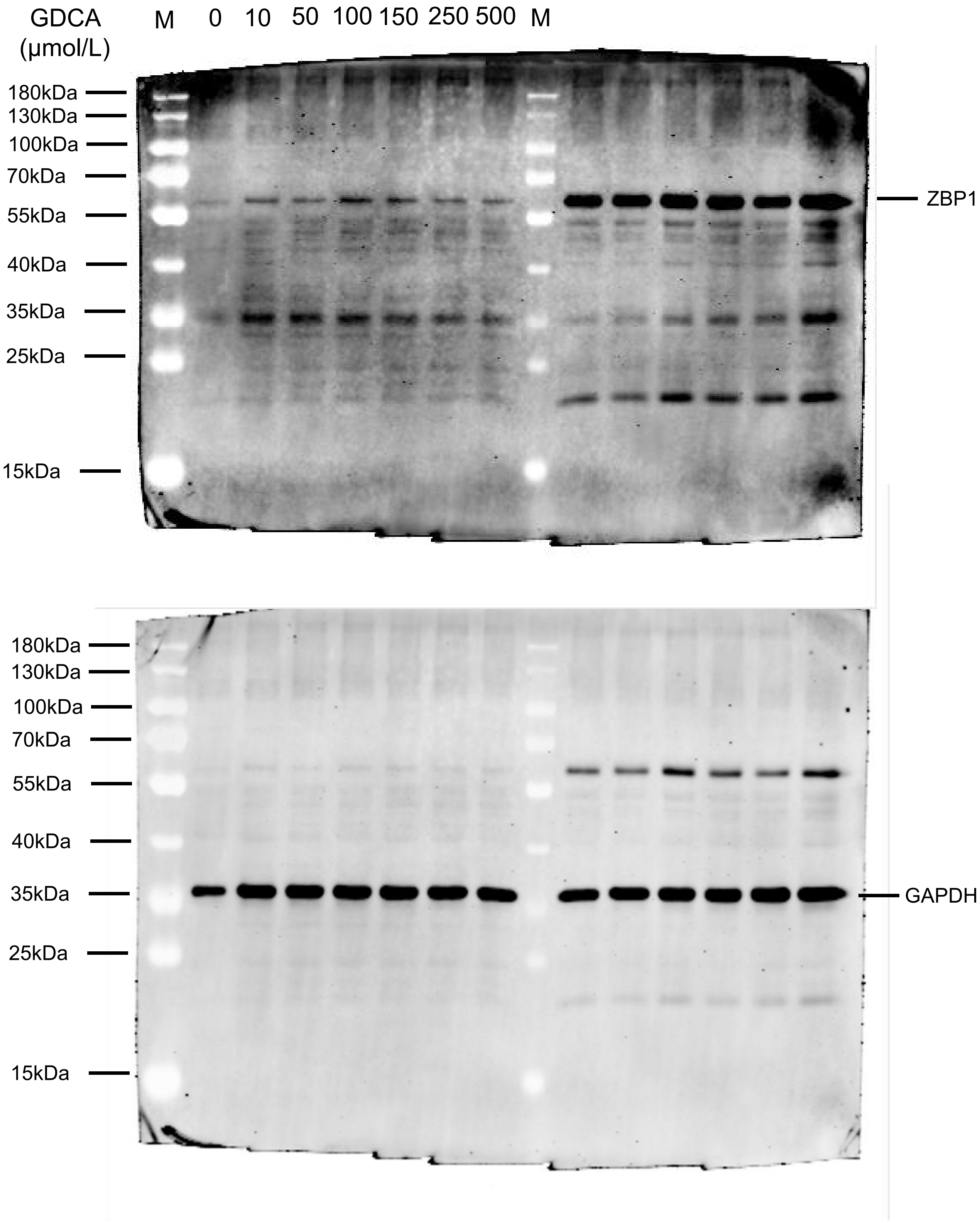

GDCA (μmol/L)
M
0
10
50
100
150
250
500
M
180kDa
130kDa
100kDa
70kDa
55kDa
40kDa
35kDa
25kDa
15kDa
ZBP1
180kDa
130kDa
100kDa
70kDa
55kDa
40kDa
35kDa
25kDa
15kDa
GAPDH

Fig. 6F

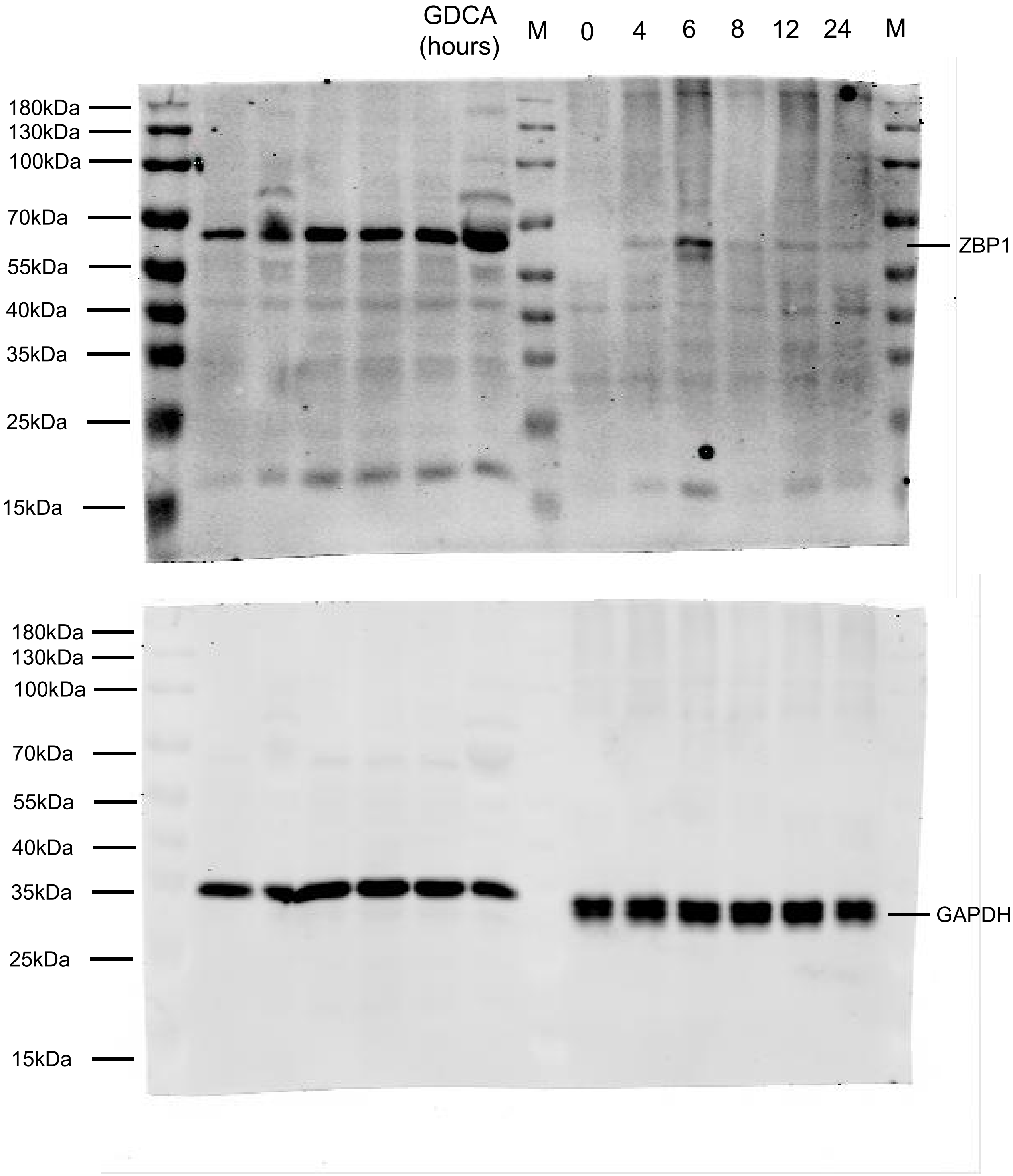
GDCA (hours)
M
0
4
6
8
12
24
M
180kDa
130kDa
100kDa
70kDa
55kDa
40kDa
35kDa
25kDa
15kDa
ZBP1
180kDa
130kDa
100kDa
70kDa
55kDa
40kDa
35kDa
25kDa
15kDa
GAPDH

Fig. 7G

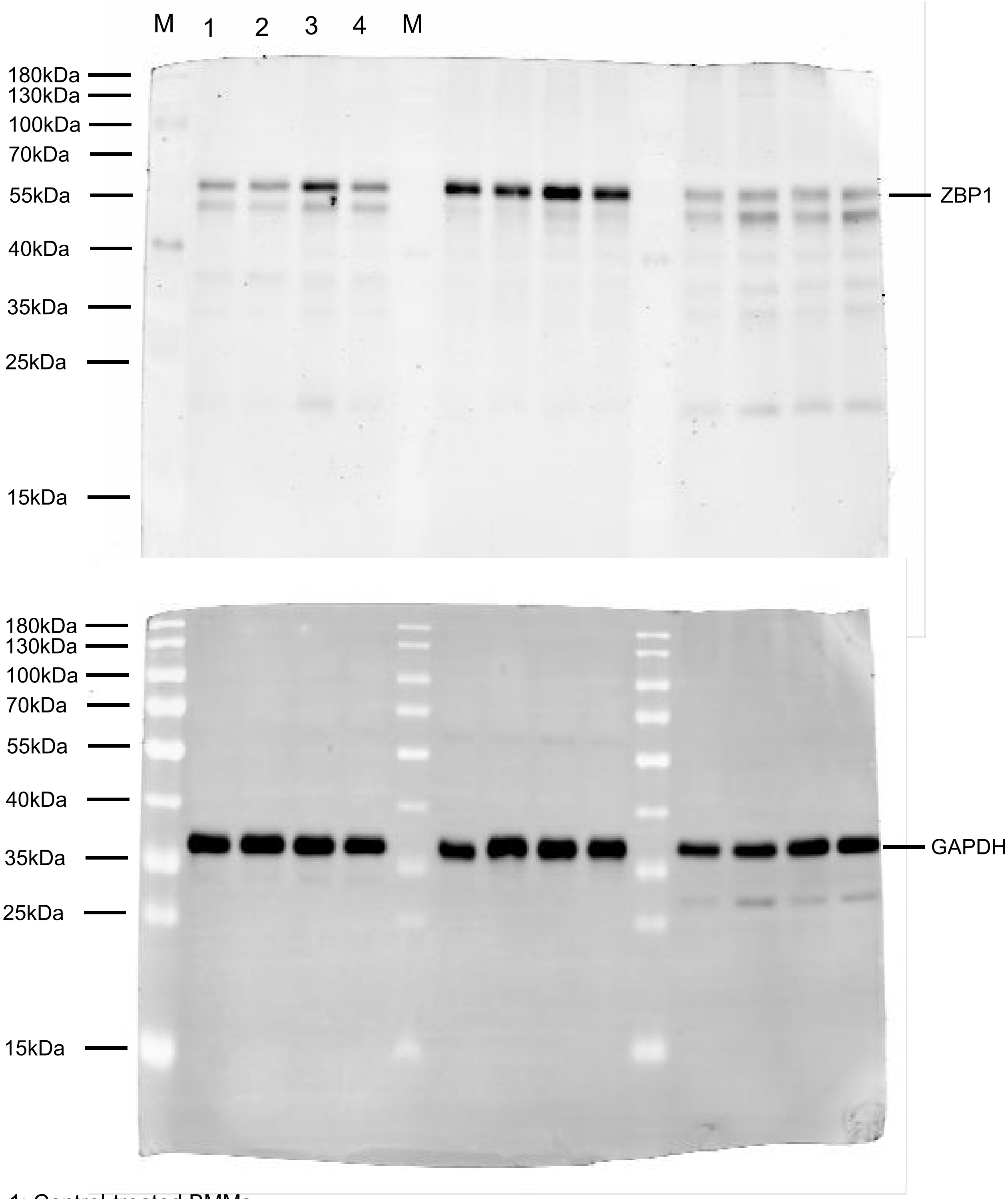

1: Control-treated BMMs
2: 10 μmol/L JTE-013-treated BMDMs
3: 100 μmol/L GDCA-treated BMMs
4: 10 μmol/L JTE-013 and 100 μmol/L GDCA-treated BMDMs

Fig. 7H

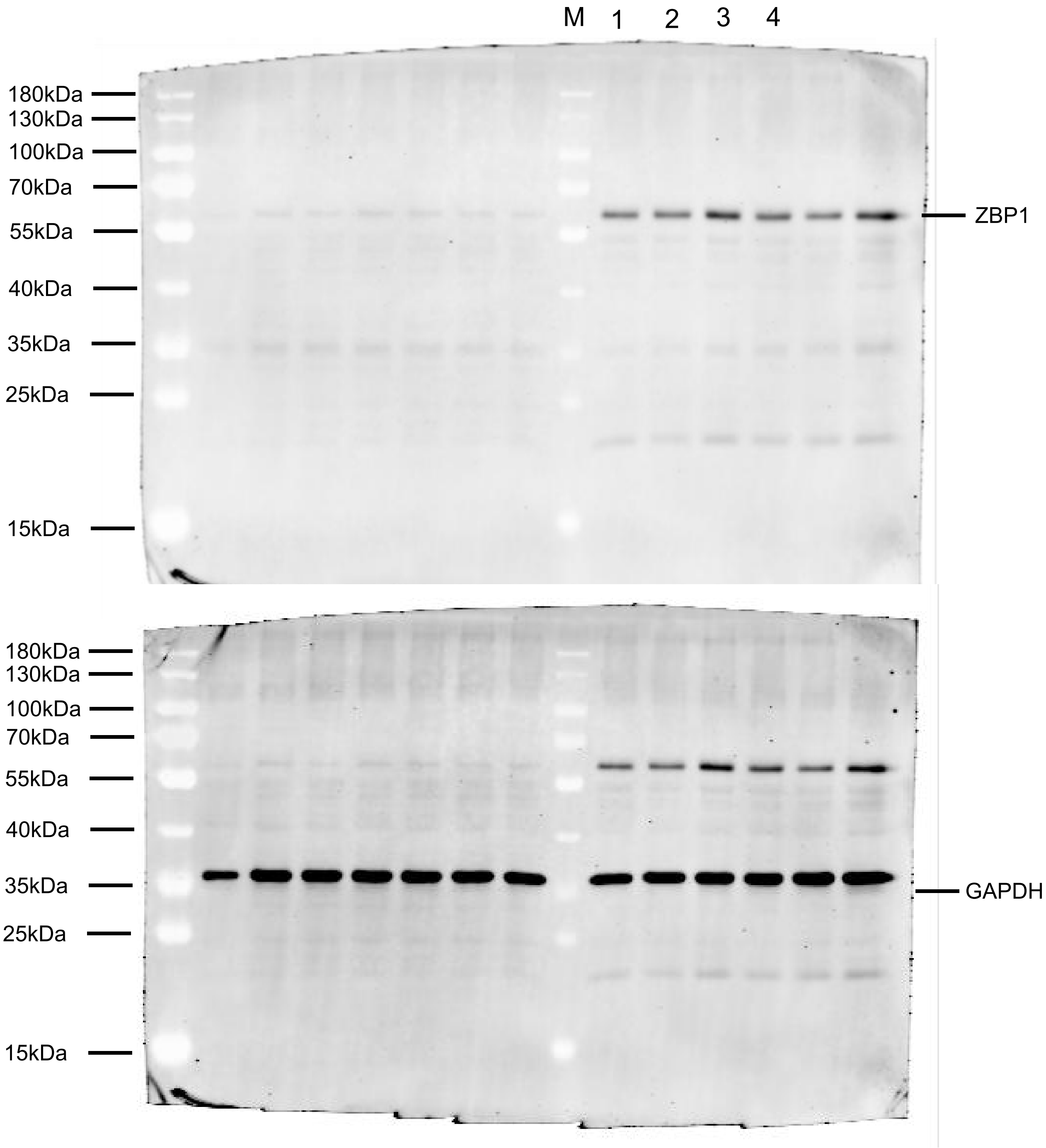

1: siNC-treated BMMs
2: siS1PR2-treated BMDMs
3: siNC and 100 μmol/L GDCA-treated BMMs
4: siS1PR2 and 100 μmol/L GDCA-treated BMDMs

Fig. 8A

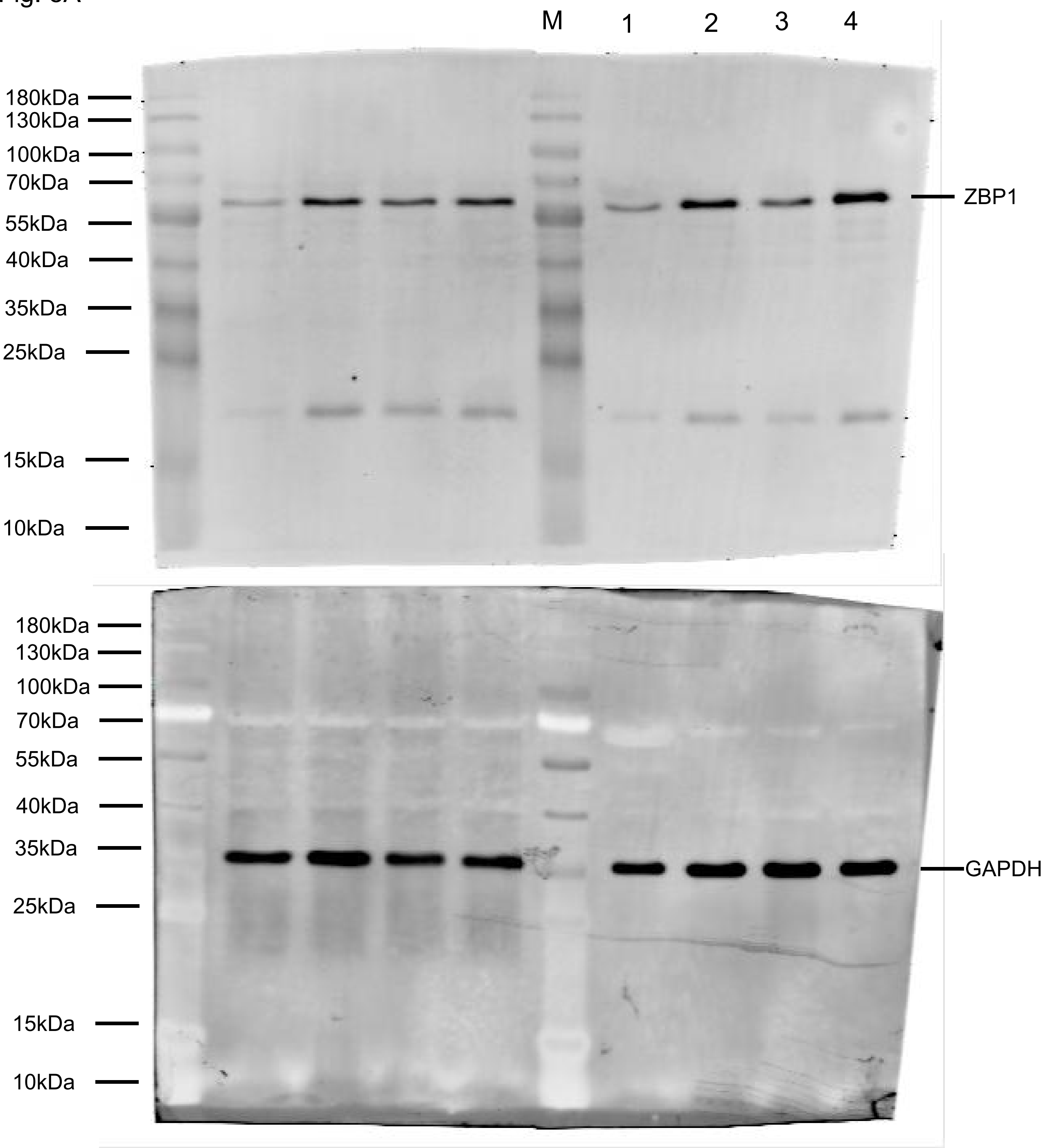

1: Control-treated BMMs
2: 100 μmol/L GDCA-treated BMDMs
3: TSZ-treated BMMs
4: TSZ and 100 μmol/L GDCA-treated BMDMs

Fig. 8A

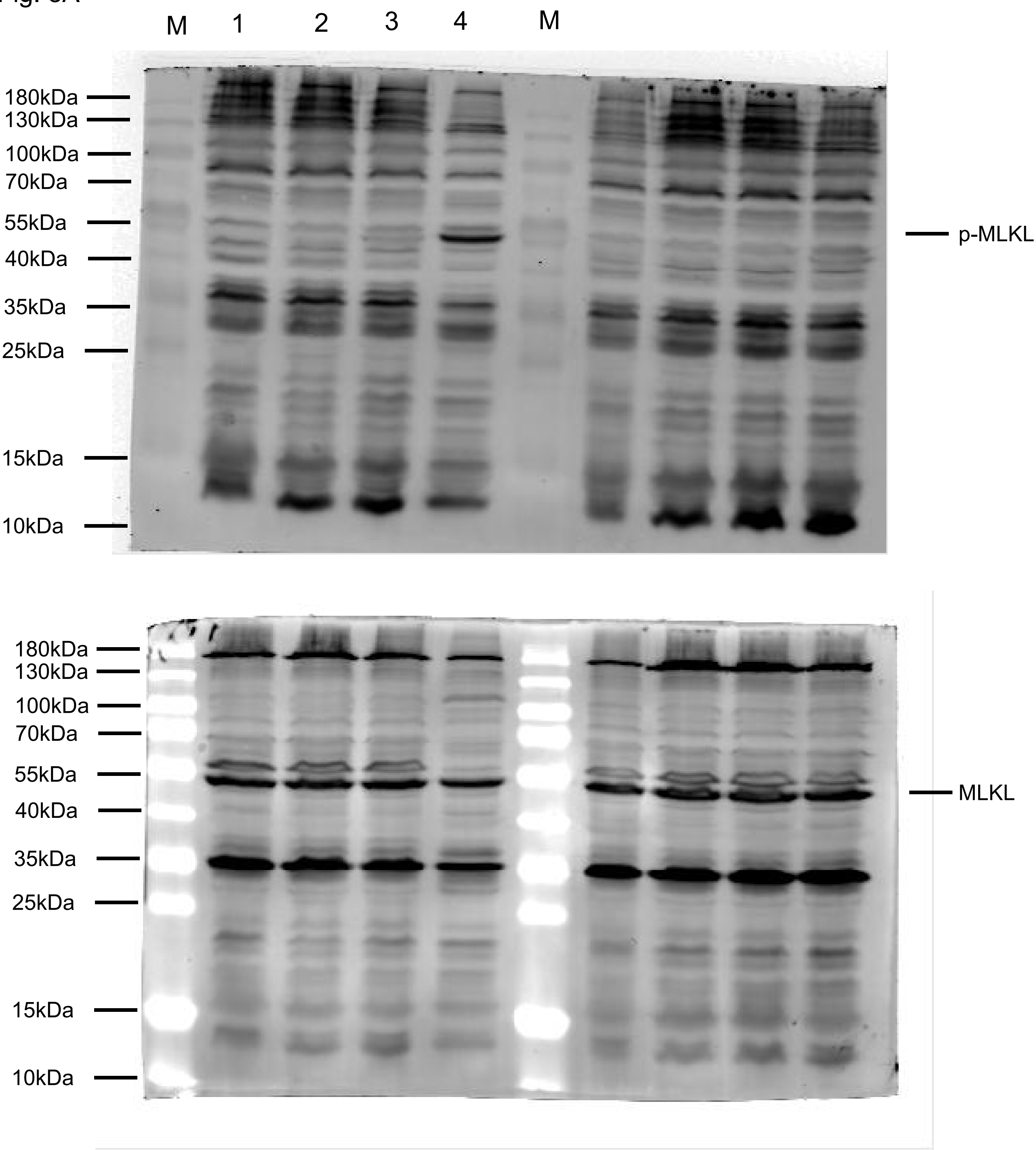

1: Control-treated BMMs
2: 100 μmol/L GDCA-treated BMDMs
3: TSZ-treated BMMs
4: TSZ and 100 μmol/L GDCA-treated BMDMs

Fig. 8C

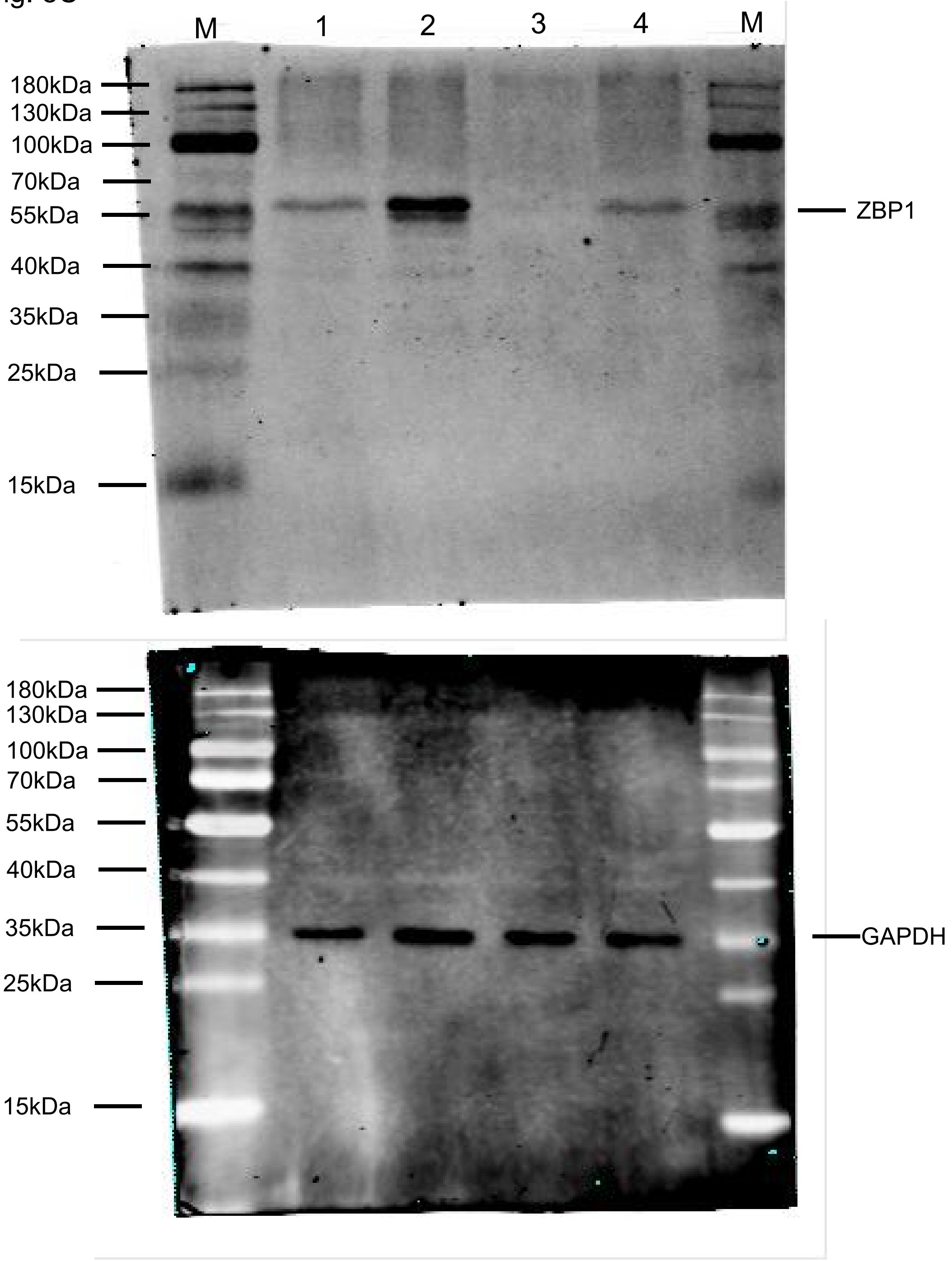

1: siNC-treated BMMs
2: siNC, TSZ, and 100 μmol/L GDCA-treated BMDMs
3: siZBP1-treated BMMs
4: siZBP1, TSZ, and 100 μmol/L GDCA-treated BMDMs

Fig. 8C

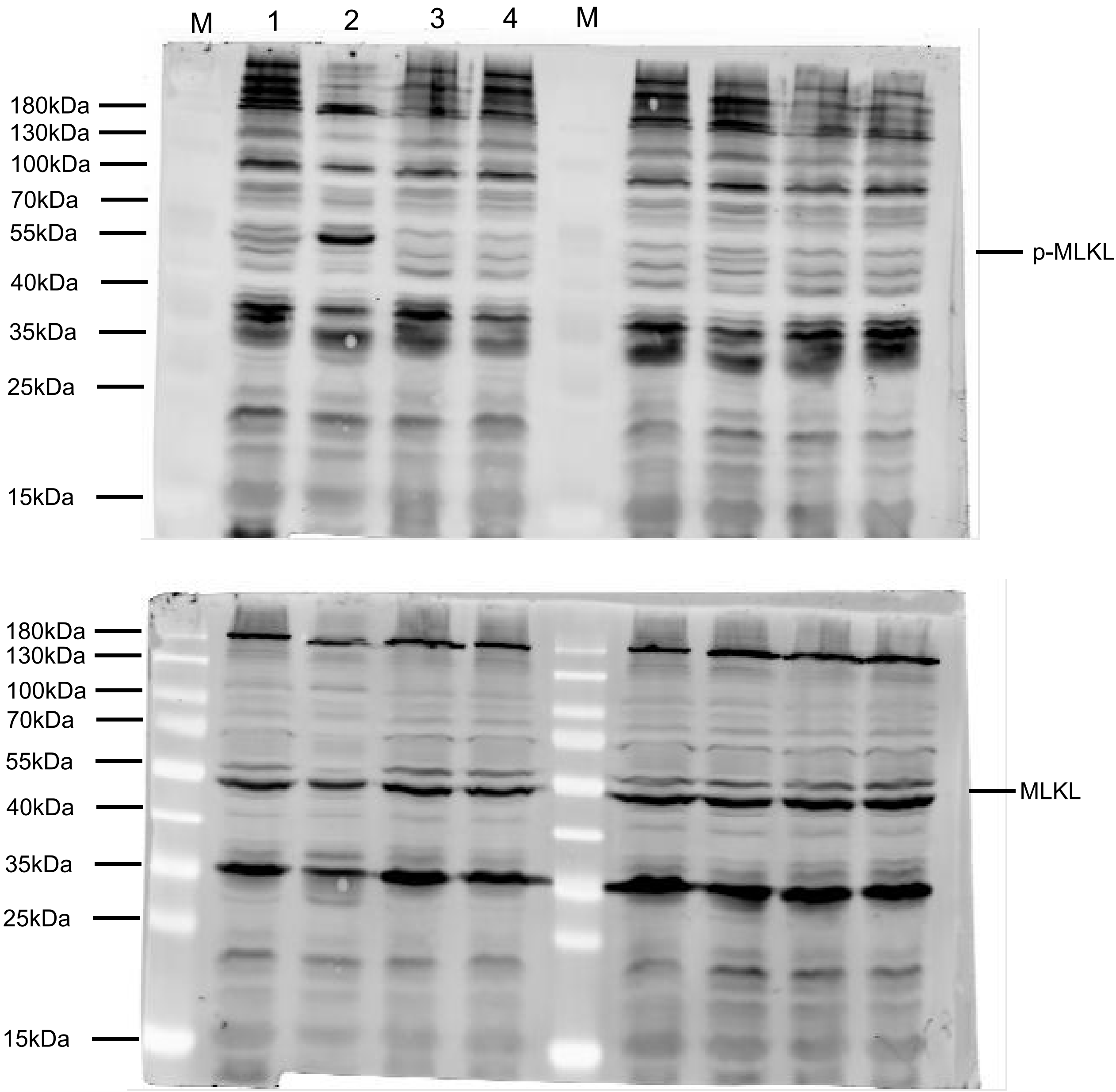

1: siNC-treated BMMs
2: siNC, TSZ, and 100 μmol/L GDCA-treated BMDMs
3: siZBP1-treated BMMs
4: siZBP1, TSZ, and 100 μmol/L GDCA-treated BMDMs

Fig. 8H

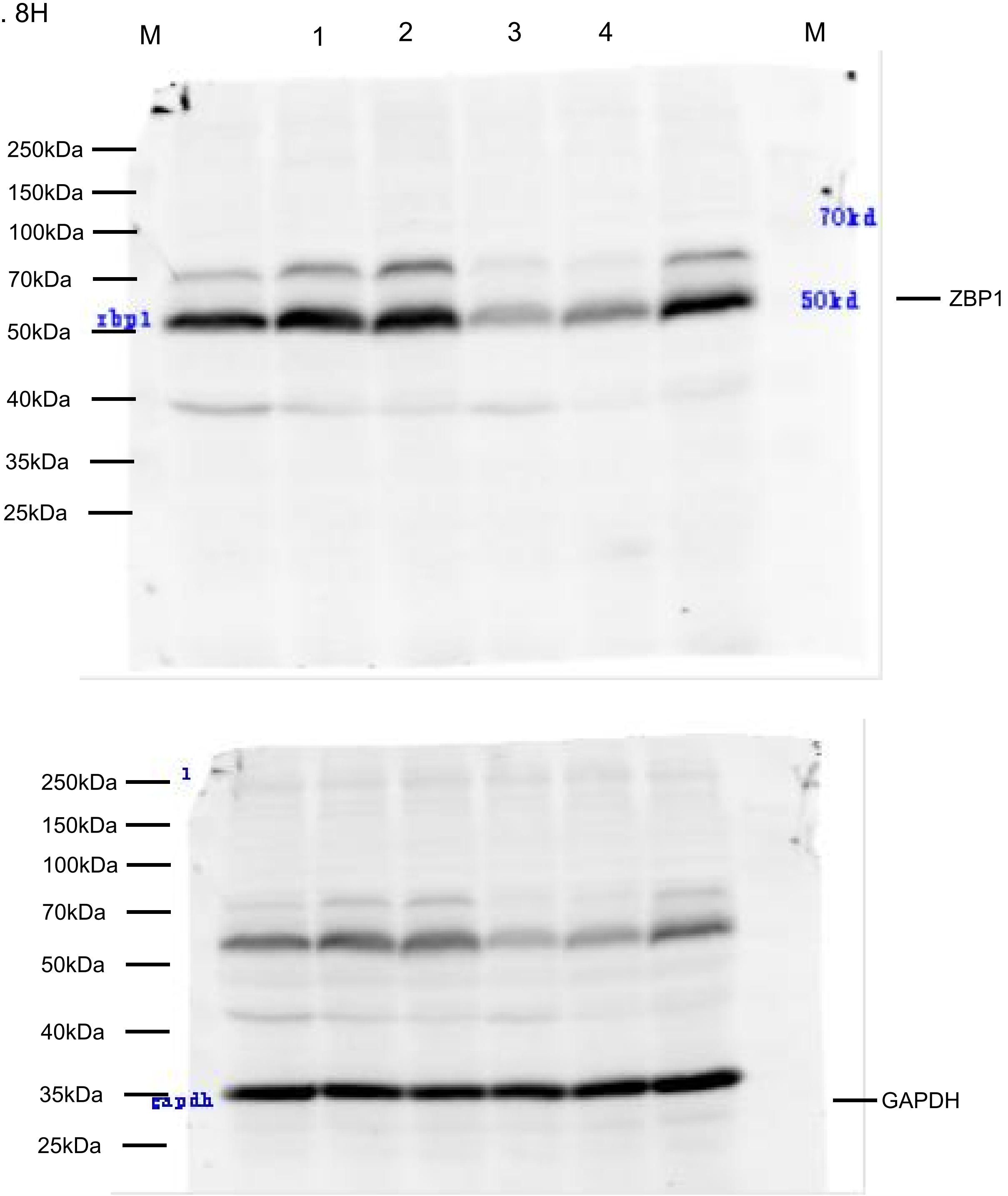

1、2: siNC-treated
3、4: siS1PR2-treated

Fig. 8H

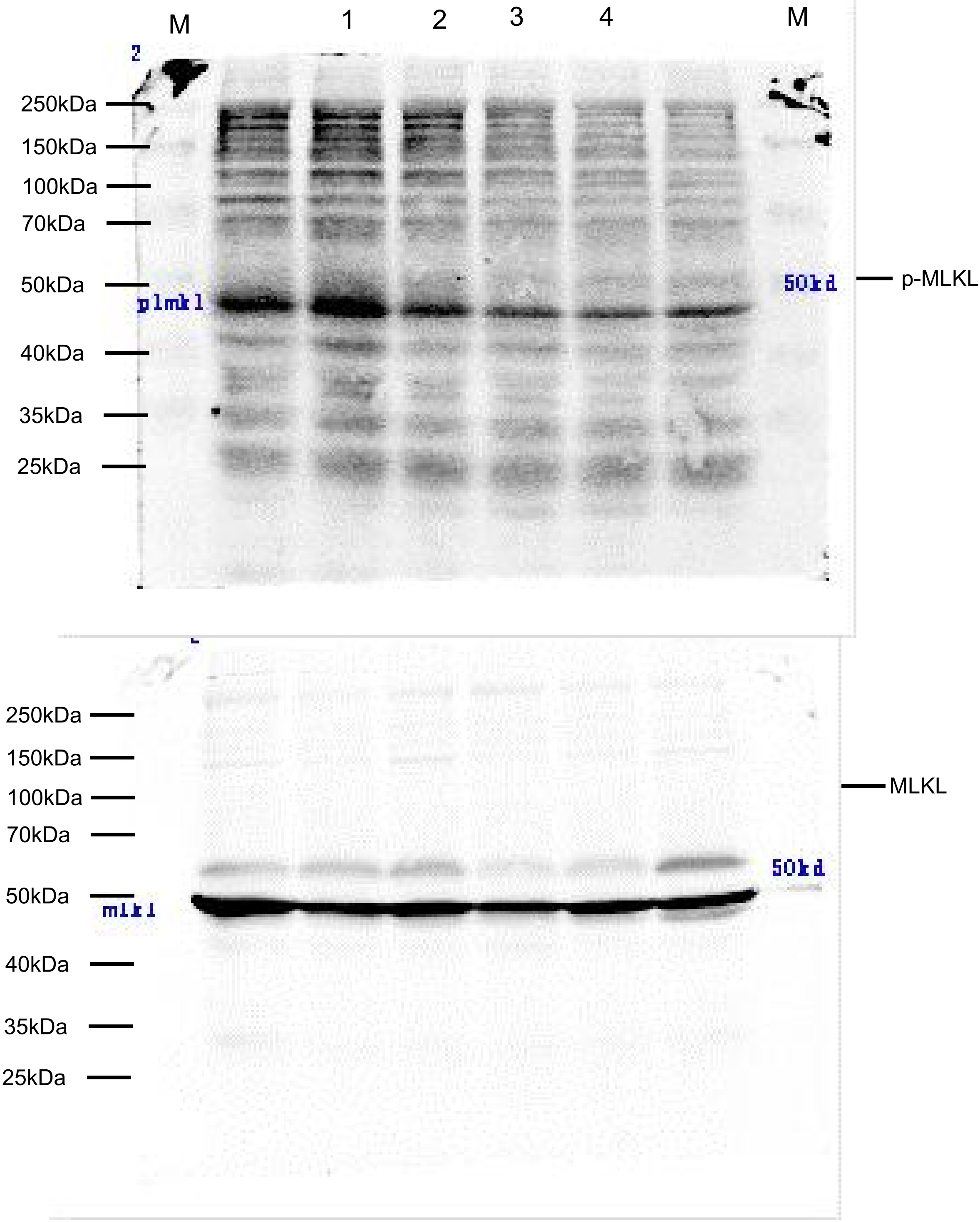

1、2: siNC-treated
3、4: siS1PR2-treated

Fig. 8H

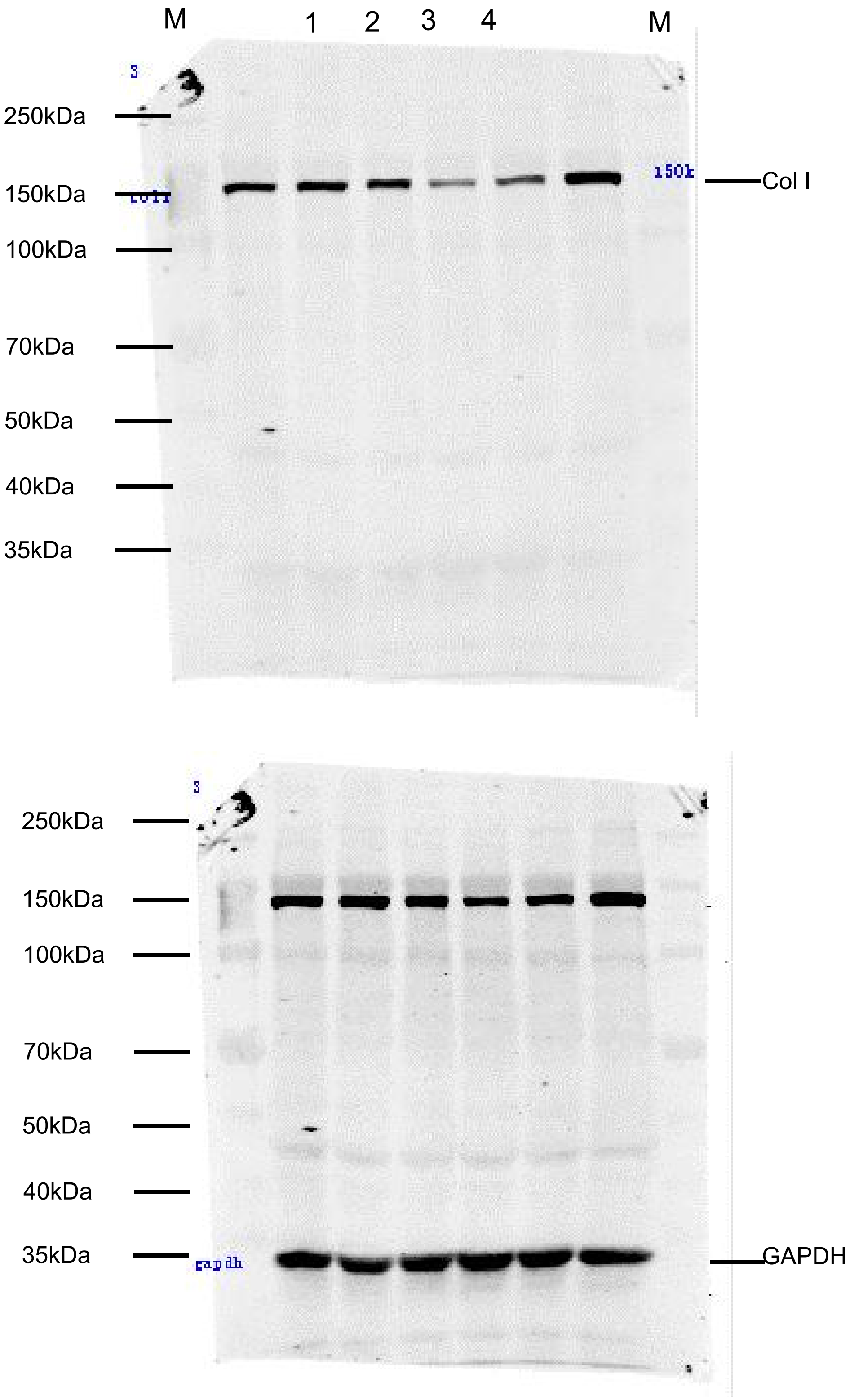

1、2: siNC-treated

3、4: siS1PR2-treated

Fig. 9F

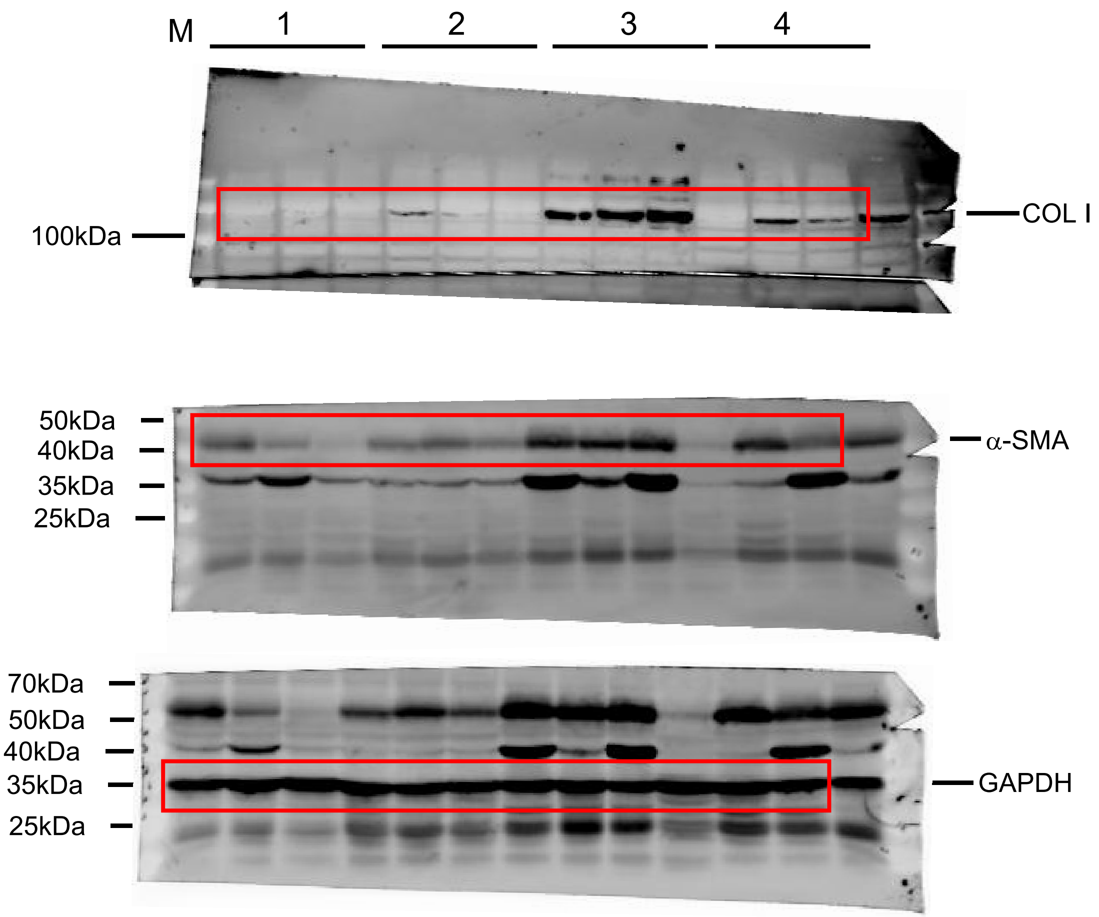

1: Sham + Nc siRNA-GeRPs
2: Sham + *Zbp1* siRNA-GeRPs
3: BDL+ Nc siRNA-GeRPs
4: BDL + *Zbp1* siRNA-GeRPs

Fig. 10F

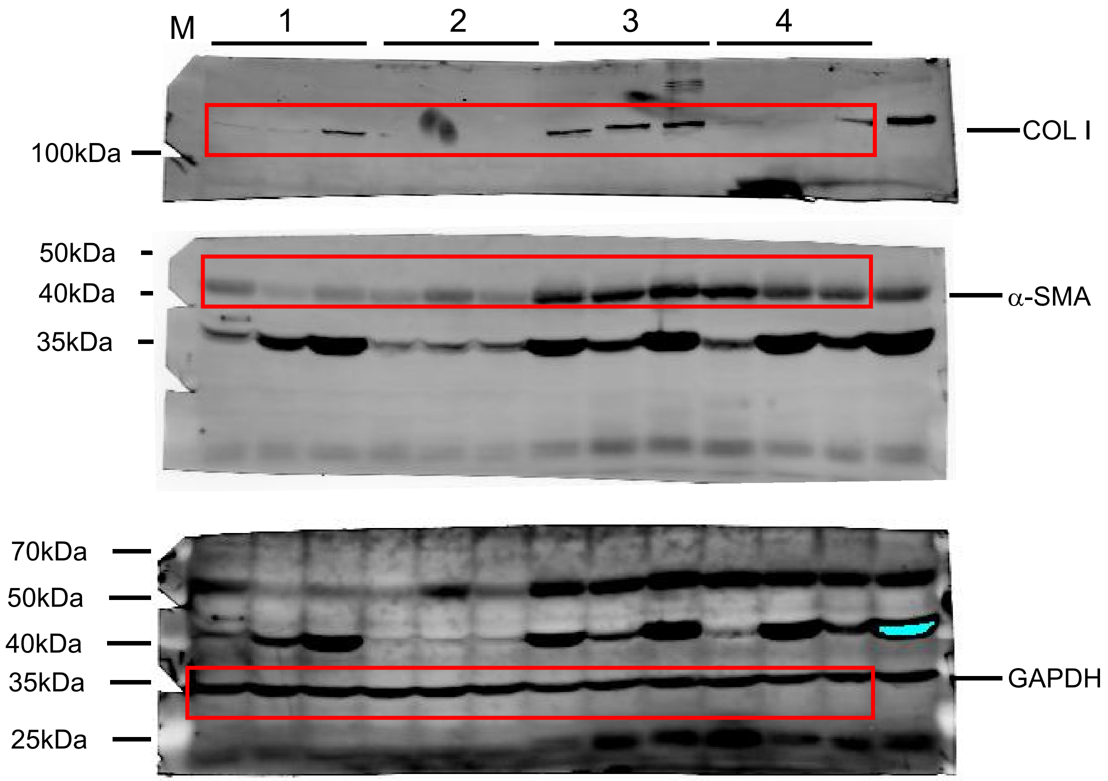

1: Sham + Nc siRNA-GeRPs
2: Sham + *Mlkl* siRNA-GeRPs
3: BDL+ Nc siRNA-GeRPs
4: BDL + *Mlkl* siRNA-GeRPs

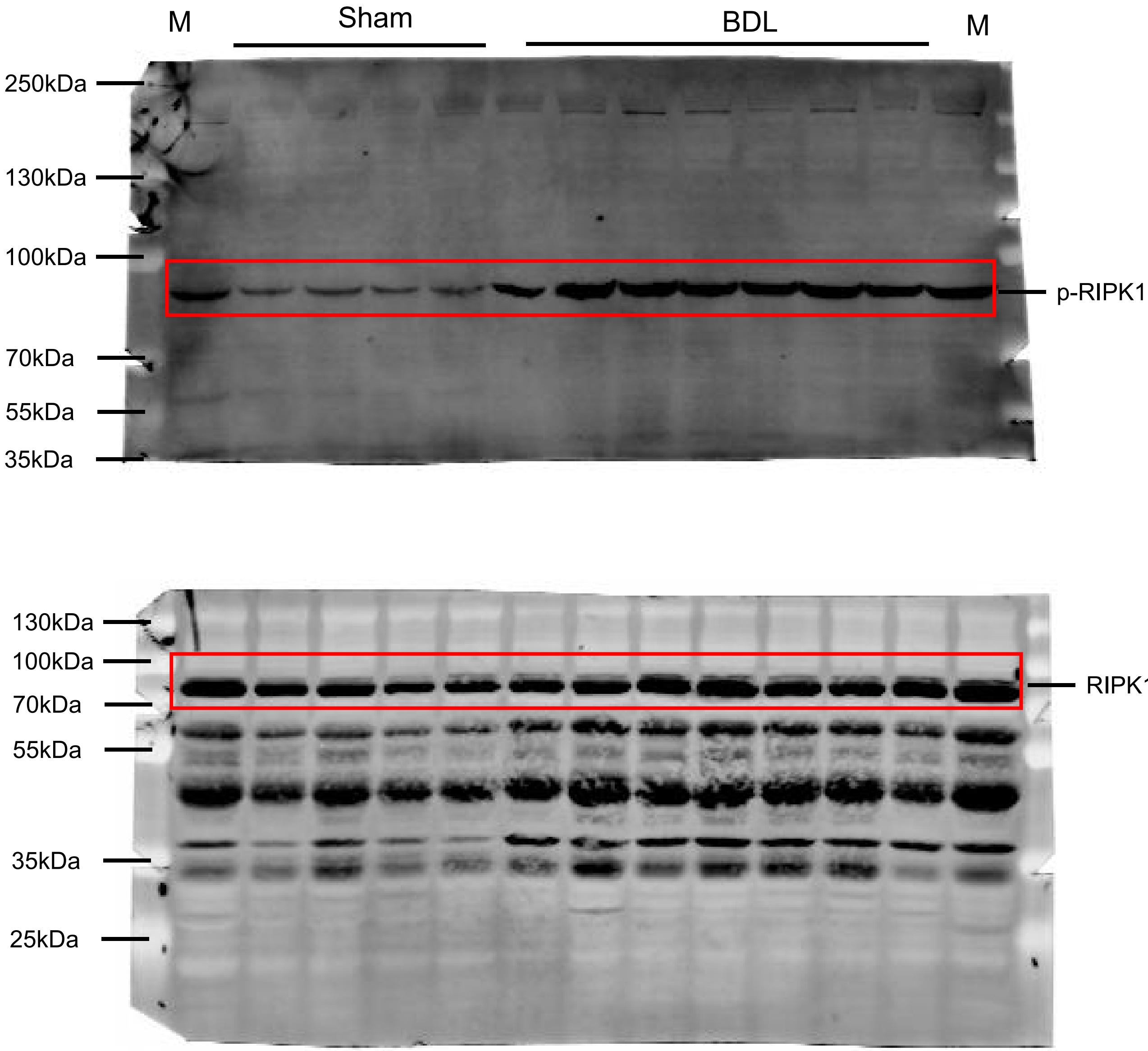

Supplementary Fig. 1B

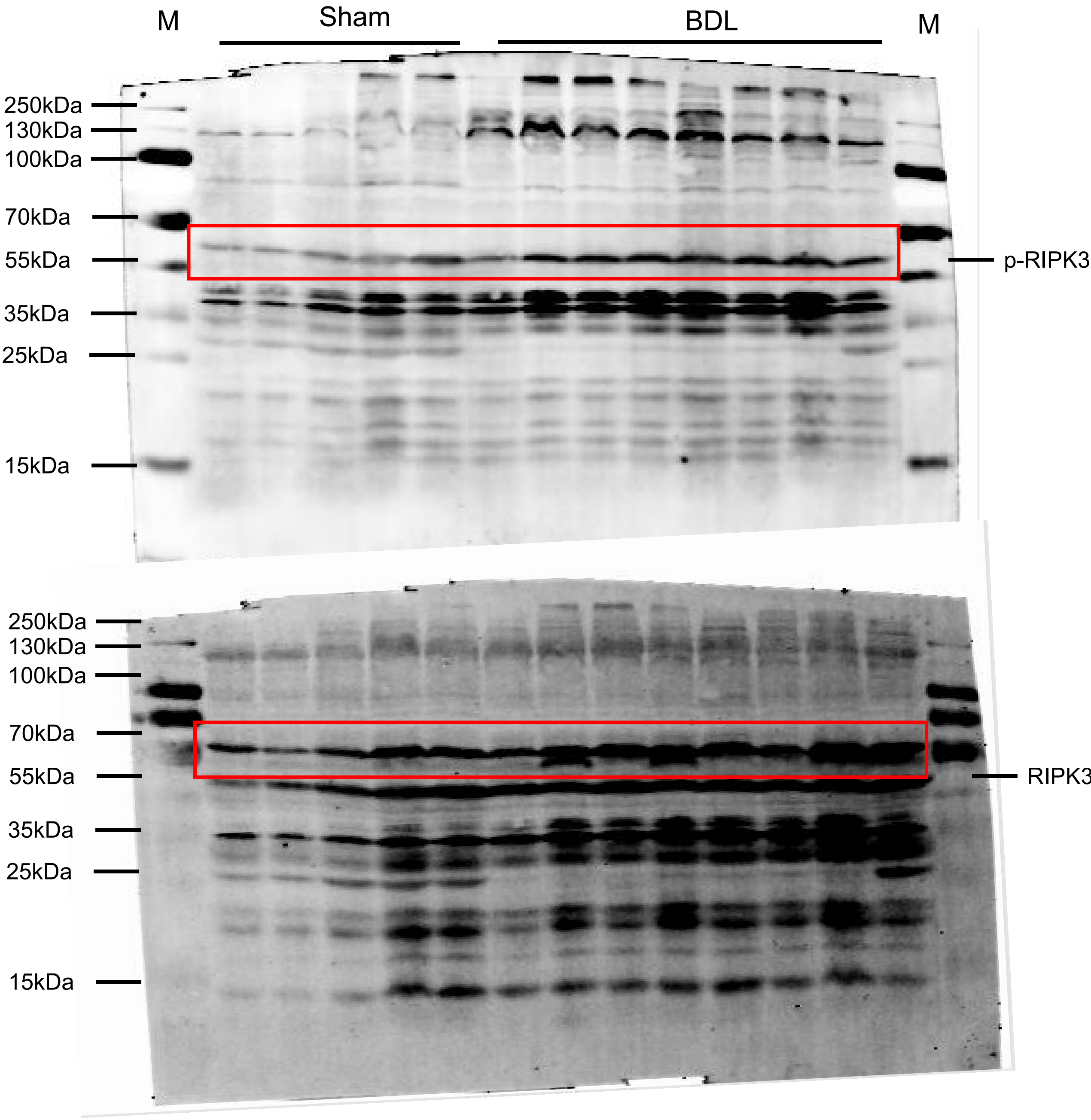

Supplementary Fig. 1B

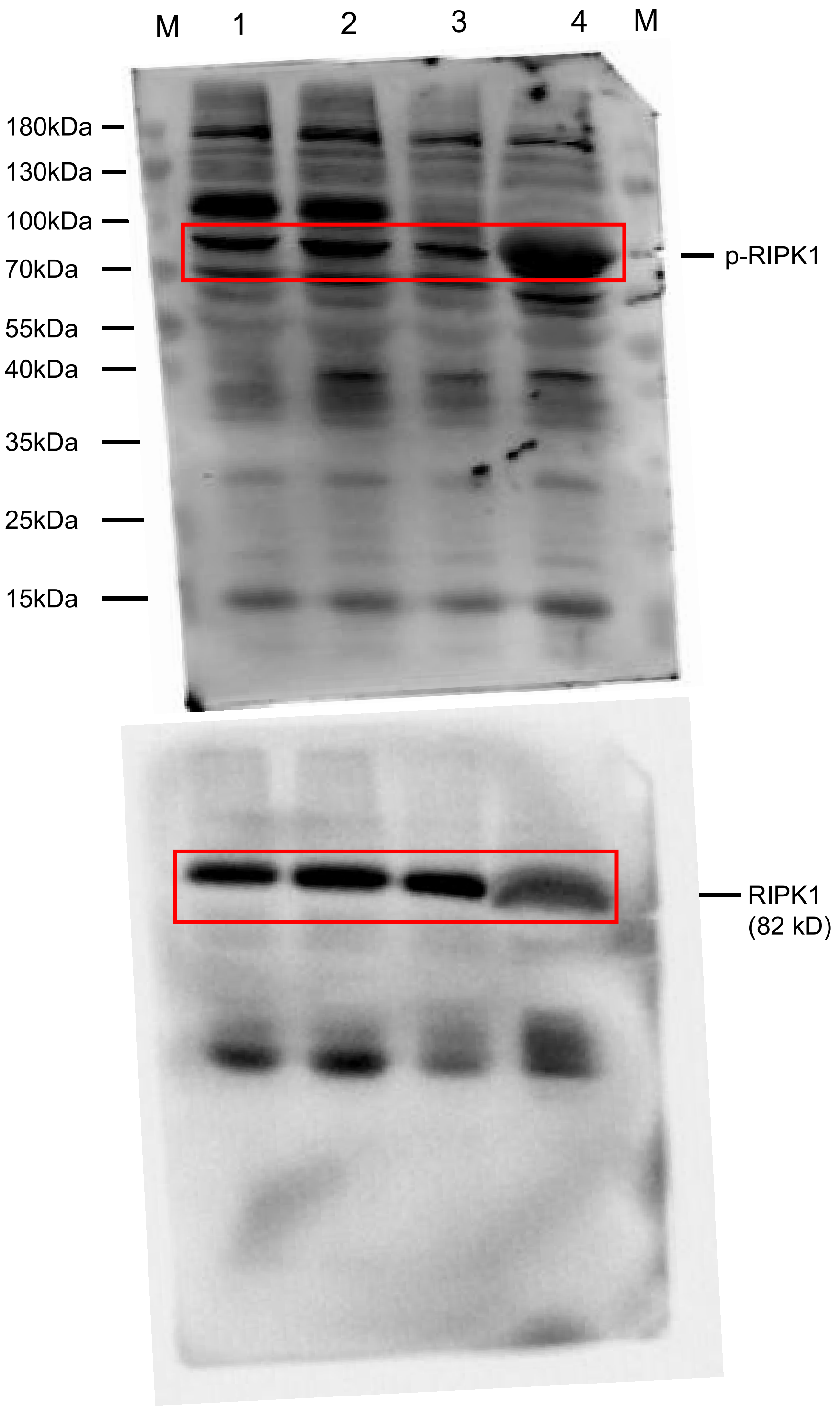

1: Control-treated BMMs
2: 100 μmol/L GDCA-treated BMDMs
3: TSZ-treated BMMs
4: TSZ and 100 μmol/L GDCA-treated BMDMs

Supplementary Fig. 1C

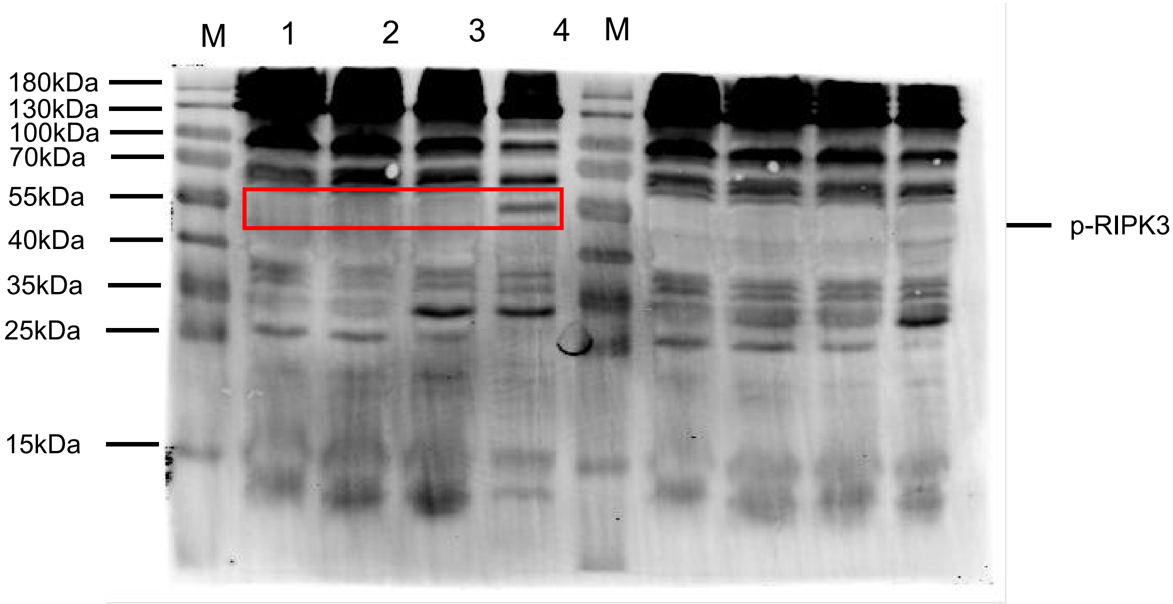

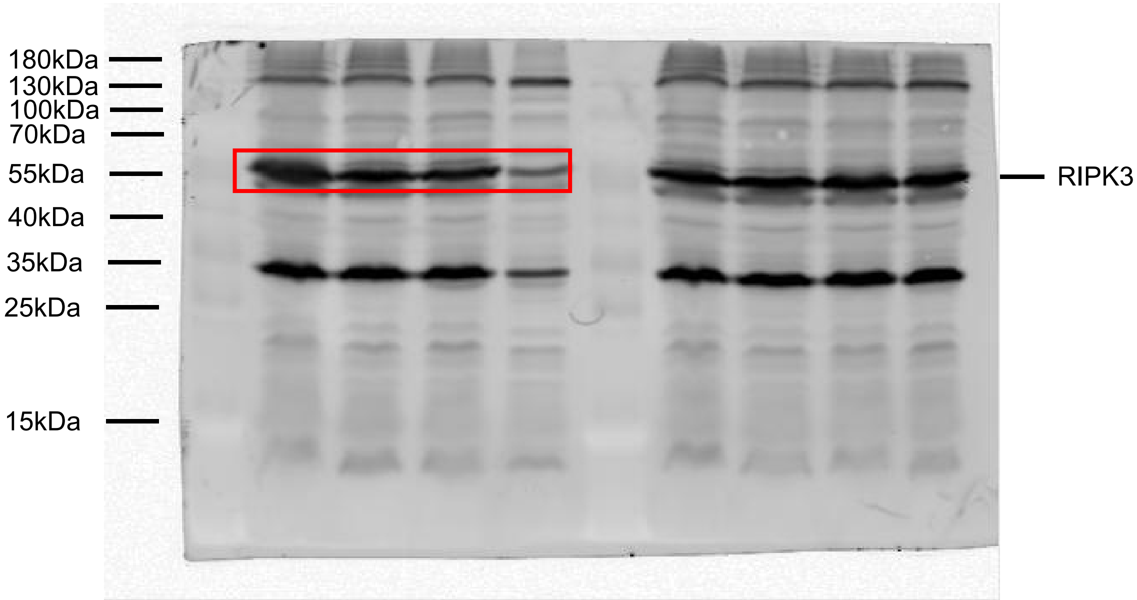

1: Control-treated BMMs
2: 100 μmol/L GDCA-treated BMDMs
3: TSZ-treated BMMs
4: TSZ and 100 μmol/L GDCA-treated BMDMs

Supplementary Fig. 1C

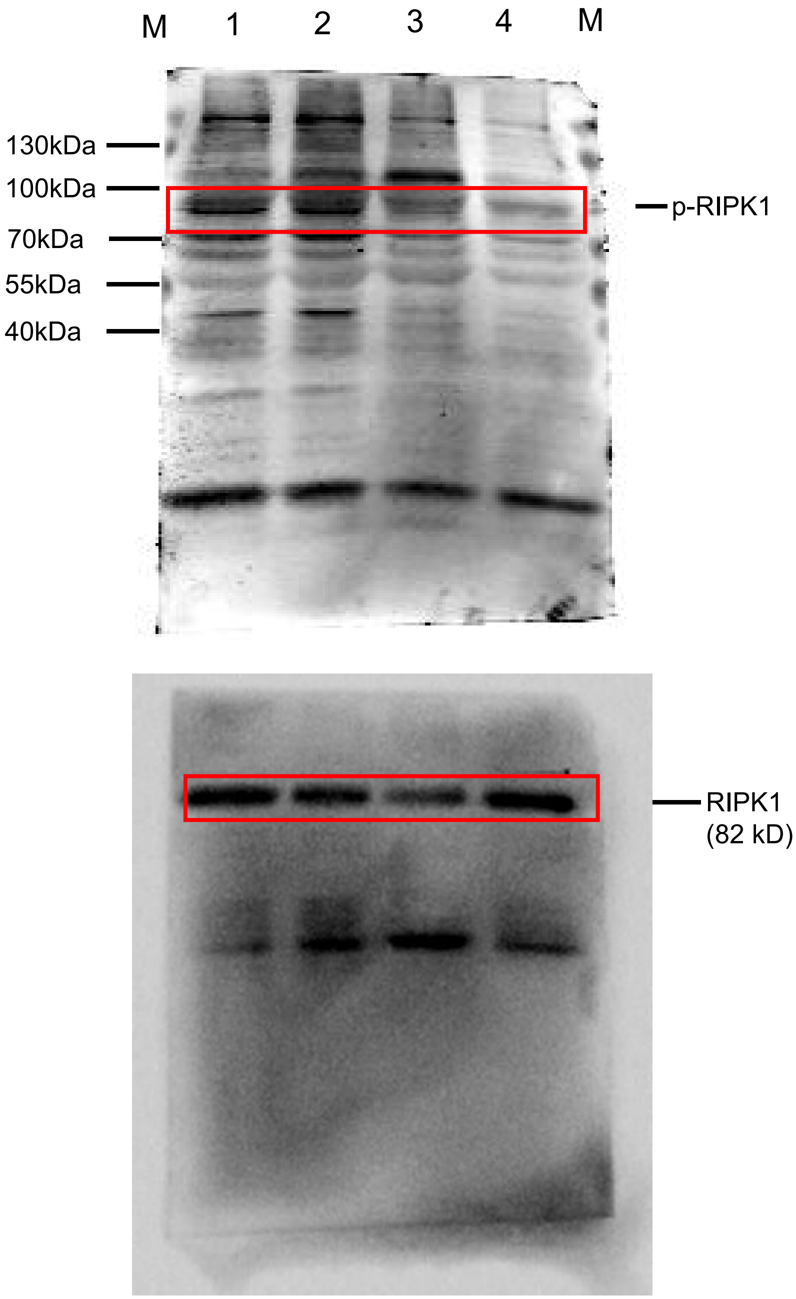

1: siNC-treated BMMs
2: siNC, TSZ, and 100 μmol/L GDCA-treated BMDMs
3: siZBP1-treated BMMs
4: siZBP1, TSZ, and 100 μmol/L GDCA-treated BMDMs

Supplementary Fig. 1D

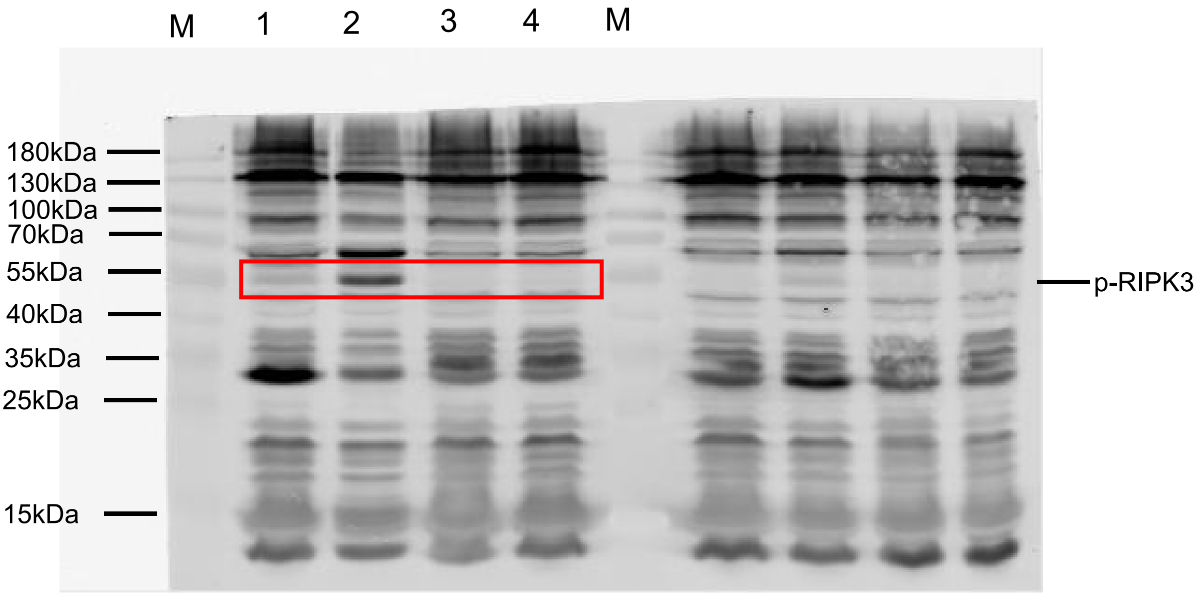

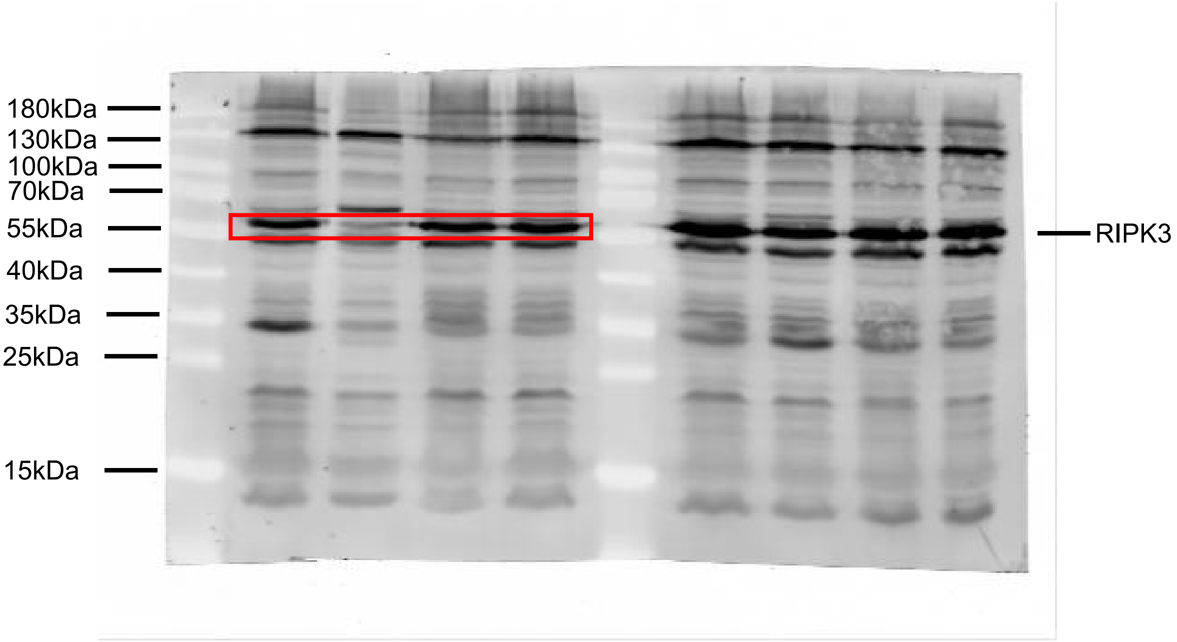

1: siNC-treated BMMs
2: siNC, TSZ, and 100 μmol/L GDCA-treated BMDMs
3: siZBP1-treated BMMs
4: siZBP1, TSZ, and 100 μmol/L GDCA-treated BMDMs

Supplementary Fig. 1D

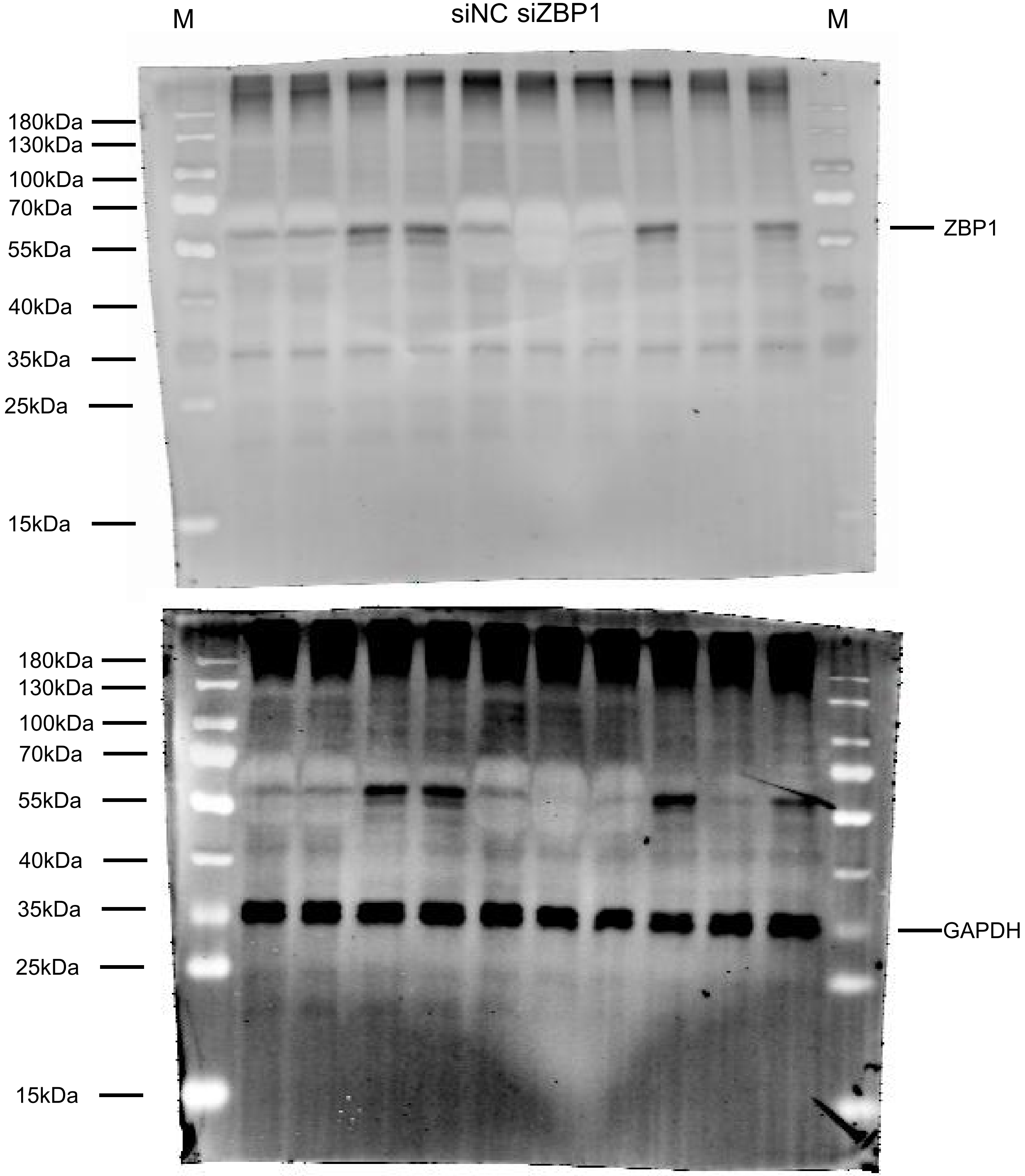

Supplementary Fig. 2B

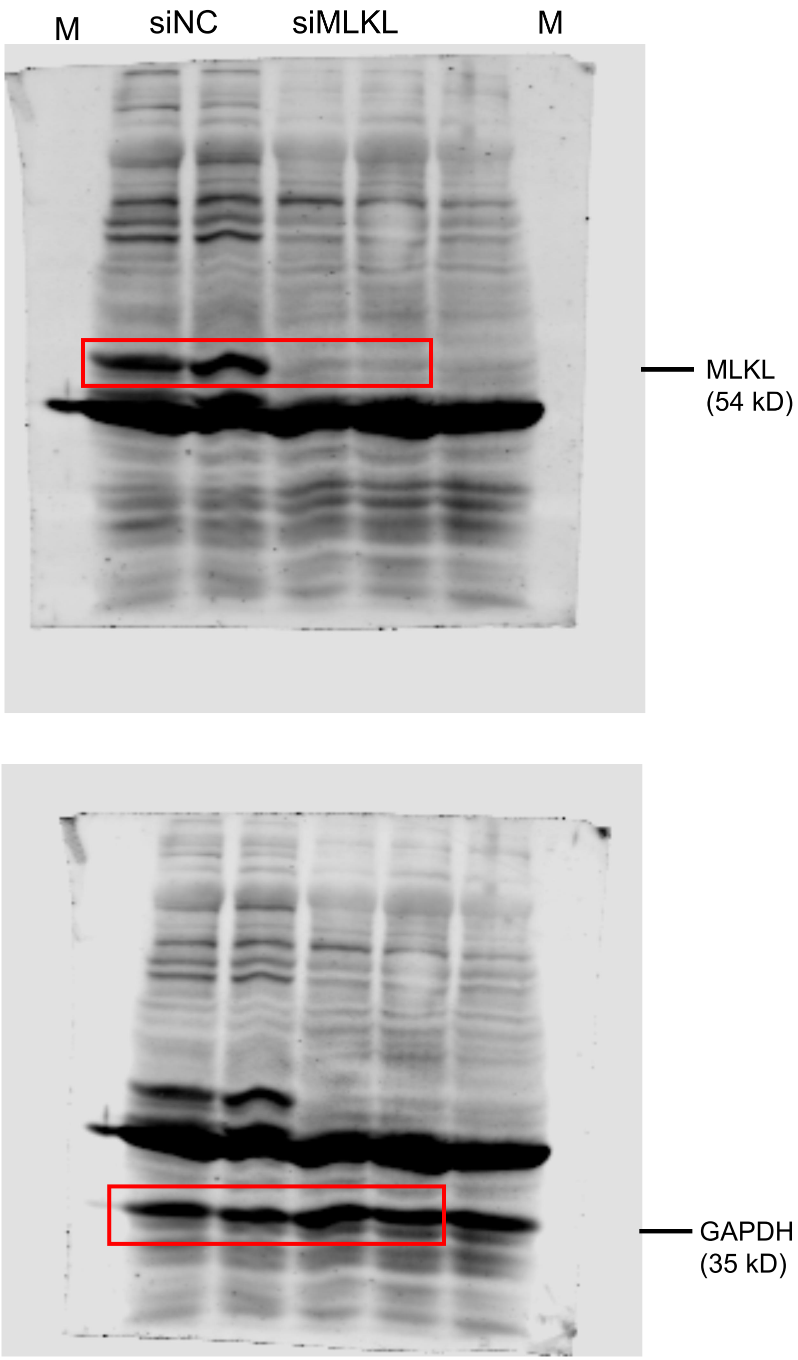

Supplementary Fig. 2C

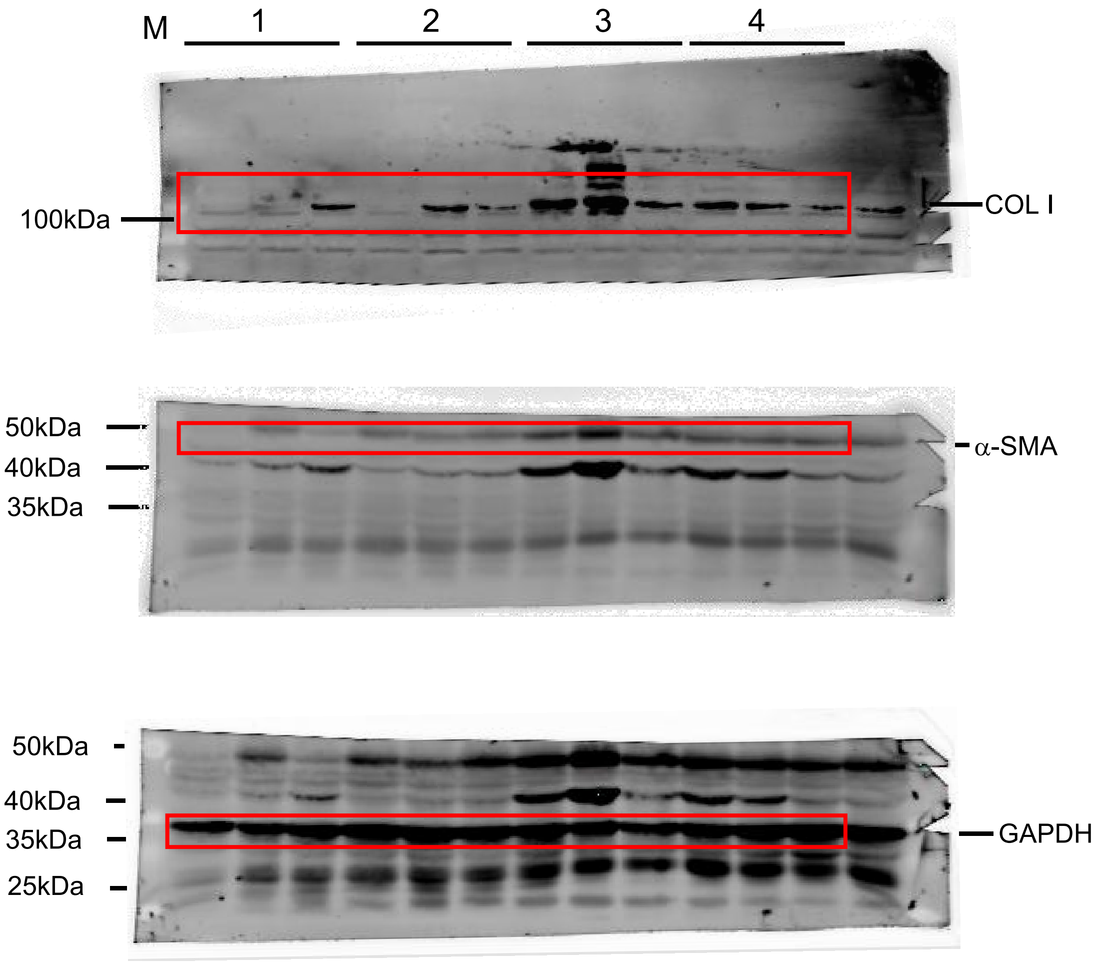

1: Sham + Vehicle
2: Sham + NSA
3: BDL + Vehicle
4: BDL + NSA

Supplementary Fig. 3E
